# Two new species and four new host records of *Fusarium* species (Nectriaceae, Hypocreales) associated with *Semanotus bifasciatus* causing *Taxodium* hybrid ‘Zhongshanshan’ dieback

**DOI:** 10.3897/mycokeys.130.177103

**Published:** 2026-04-06

**Authors:** Jiao He, Ning Li, De-Wei Li, Ying Guo, Shan-Shan Chen, Feng Sun, Jun-Wei Wang, Lin Huang

**Affiliations:** 1 Co-Innovation Center for Sustainable Forestry in Southern China, Nanjing Forestry University, Nanjing, Jiangsu 210037, China Co-Innovation Center for Sustainable Forestry in Southern China, Nanjing Forestry University Nanjing China; 2 The Connecticut Agricultural Experiment Station Valley Laboratory, Windsor, CT 06095, USA The Connecticut Agricultural Experiment Station Valley Laboratory Windsor United States of America; 3 Lianhua Forest Farm, Department of Natural Resources and Forestry & Grassland, Banma County, Guoluo, Qinghai 814300, China Lianhua Forest Farm, Department of Natural Resources and Forestry & Grassland Banma County China; 4 Jinan State-Owned Beijiao Forest Farm, Jinan, Shandong 250119, China Jinan State-Owned Beijiao Forest Farm Jinan China; 5 Jingjiang National Forest Tree Improved Variety Base of Taxodium hybrid ‘Zhongshanshan’, Jingjiang, Jiangsu 214500, China Jingjiang National Forest Tree Improved Variety Base of Taxodium hybrid ‘Zhongshanshan’ Jingjiang China; 6 Kunyushan Forest Farm, Yantai, Shandong 264100, China Kunyushan Forest Farm Yantai China

**Keywords:** Ascomycota, Cerambycidae, Cupressaceae, fungal diversity, pathogenic fungi, quarantine wood-boring pest

## Abstract

*Semanotus bifasciatus* (Motschulsky) (Cerambycidae, Coleoptera) is a quarantine wood-boring pest that severely damages cypress trees in China and poses a significant threat to forest ecological security. However, the knowledge of *Fusarium* species associated with this beetle is inadequate in China. In this study, 16 strains of *Fusarium* were isolated from beetle galleries in infected *Taxodium* hybrid ‘Zhongshanshan’ samples. Morphological and molecular multi-locus analyses, based on internal transcribed spacer region of the translation elongation factor 1-alpha (*TEF-1α*), RNA polymerase largest subunit (*RPB1*) and RNA polymerase second largest subunit (*RPB2*) genes, identified four new host records species (*F. annulatum*, *F. fujikuroi*, *F. ipomoeae* and *F. oblongum*) and two new species (*F. semanoti***sp. nov**. and *F. taxodii***sp. nov**.) are introduced in the present study, with pathogenicity tests confirming all six species could cause *T.* hybrid ‘Zhongshanshan’ dieback. This study provides the first documentation of *Fusarium* diversity associated with *S. bifasciatus* in China, offering new perspectives for understanding the beetle-fungus symbiotic system and their synergistic pathogenicity to *T.* hybrid ‘Zhongshanshan’.

## Introduction

*Semanotus bifasciatus* (Motschulsky) (Cerambycidae, Coleoptera), commonly known as the double-striped cedar borer (Xiao 1992), is one of the most important stem-boring forest pests in China (Gao et al. 2007). Its larvae damage the stems of Cupressaceae trees species, such as *Platycladus orientalis* (L.) Franco, *Sabina chinensis* (L.) Antoine, *Thujopsis dolabrata* (L.f.) Siebold & Zucc. and *Juniperus rigida* Siebold & Zucc. In Taian, Shandong Province, it has also been found to infest *Sophora japonica* L. (Gu et al. 1997). It is widely distributed across numerous provinces and municipalities in China. In 1996, the former Ministry of Forestry designated it as a forest plant quarantine pest. In 2013, it was included in the National List of Forestry Dangerous Pests by the former State Forestry Administration and has also been listed as a supplementary forestry quarantine pest in Anhui, Guizhou, Hebei, Henan, Liaoning, Shaanxi, Shanghai, Shanxi and Tianjin (Li et al. 2025). In recent years, *S. bifasciatus* has been severely infesting *P. orientalis* in multiple Chinese regions, thereby posing a severe threat to ancient cypress trees, forest resources and the ecological environment. Notable examples include an infection rate of 97% on *P. orientalis* in Beijing (1979) and about 90% on dead *P. orientalis* in Shandong (2002–2006) (Zhang et al. 2019), as well as 67.3% on afforestation seedlings in Liaoning (1988) (Wang 1996) and 88.2% on junipers in Jiangsu (Shen et al. 2001; Hu 2003). Additionally, in 2018, *S. bifasciatus* severely affected the growth, ecology and economic value of trees in the mountain forests in Jinan’s suburban area. It killed over 2,000 Chinese junipers, incurring a direct economic loss of ¥162,000 yuan (about $25,100 US dollars) (Fu et al. 2021).

*Taxodium* hybrid ‘Zhongshanshan’ (also known as, Nanjing Beauty) is a superior interspecies hybrid of *T. distichum* (L.) Rich. and *T. mucronatum* Ten. (Zhu 2017). Developed through years of experimental research by the Nanjing Botanical Garden, Chinese Academy of Sciences, it is a national-level superior tree species for landscaping in China (Qi 2018). Possessing prominent traits of moisture tolerance, decay resistance, salt-alkali tolerance and good adaptability to degraded lands, makes *T.* hybrid ‘Zhongshanshan’ a highly versatile tree species. It plays a crucial role in landscaping, ecological conservation, littoral zone afforestation and development of forest cities (Tang and Yin 2016; Zhu 2017; Qi 2018). *Taxodium* hybrid ‘Zhongshanshan’ is widely planted in south-eastern China. Large-scale artificial ecological forests of *T.* hybrid ‘Zhongshanshan’ have been established in the Yangtze River Basin, with a total of plants over 50 million (Shi et al. 2022b). Studies indicate that the carbon storage of existing *T.* hybrid ‘Zhongshanshan’ stands in the Yangtze River Basin can reach 217 tonnes per hectare (Shi et al. 2022b, 2022a). Given that most stands are not yet mature and afforestation areas continue to expand, its carbon sequestration potential is enormous. The demand for landscape plantations and timber for this species still outstrips supply (Shi et al. 2022b). Notably, no severe diseases or pests were recorded in *T.* hybrid ‘Zhongshanshan’ over more than 20 years of afforestation and promotion. However, in this study, severe dieback of this tree species was first observed in Shandong Province in 2024.

The genus *Fusarium* (Nectriaceae) is one of the most renowned genera that contains endophytes, saprotrophs and phytopathogens (Link 1809). The members of this genus can directly incite diseases in plants, humans and domesticated animals (Rabodonirina et al. 1994; Boonpasart et al. 2002; Vismer et al. 2002). *Fusarium* was recently included in the top 10 globally most important genera of plant pathogenic fungi, based on scientific and economic importance (Dean et al. 2012). The members of the *F. fujikuroi* species complex (FFSC) are well known for their abilities to cause devastating diseases, such as rice bakanae, maize ear rot and soybean root rot, leading to considerable reductions in crop yields and income (O’Donnell et al. 2015; Qiu et al. 2020). The members of the *F. solani* species complex (FSSC) cause plant diseases, mostly root and crown rots and vascular wilts on a wide range of plants, including soybeans, potato, cucurbits, peas, sweet potato, Chinese rose and various legumes (Coleman 2016; Summerell 2019; He et al. 2021). Current research on *Fusarium* taxonomy has established an integrated system combining morphology and molecular systematics. Morphology remains the foundational basis for classification, with diagnostic characteristics encompassing the asexual stage (e.g. colony morphology, pigmentation, conidiophores, conidiogenous cells, macro- and microconidial morphology and the presence or absence of chlamydospores) (Leslie and Summerell 2006) and the sexual stage (e.g. characteristics of perithecia, paraphyses, asci and ascospores) (Rossman et al. 1999). However, morphological identification is limited by its susceptibility to variations in growth media and conditions, as well as high interspecies similarity, which restricts its independent discriminatory power (Crous et al. 2021). Therefore, modern *Fusarium* classification prioritises multilocus phylogenetic analyses as its core approach, adopting the Genealogical Concordance Phylogenetic Species Recognition (GCPSR) concept (Taylor et al. 2000). Commonly used molecular markers include the internal transcribed spacer region and intervening 5.8S nrRNA gene (ITS), RNA polymerase II largest subunit (*RPB1*), RNA polymerase II second largest subunit RPB*2*), β-tubulin (*TUB2*), translation elongation factor 1-alpha (*TEF1*) genes and others (Lombard et al. 2015; Sandoval-Denis et al. 2018; Crous et al. 2021). Amongst these, *TEF1* and *RPB2* are preferred as primary markers due to their high resolution and well-curated public databases (O’Donnell 2000; Crous et al. 2021). At present, GCPSR-based multigene data analyses have resolved *Fusarium* into at least 300 phylogenetically distinct species distributed amongst 23 monophyletic species complexes and several single-species lineages (O’Donnell et al. 2015; Summerell 2019; O’Donnell et al. 2020; Geiser et al. 2021).

This study aimed to identify the *Fusarium* species isolated from the larval galleries of *S. bifasciatus* using morphological characters and multi-locus phylogenetic analyses, so as to clarify the fungal diversity associated with *S. bifasciatus*. Additionally, a pathogenicity test was conducted to determine the causal agent of dieback in *T.* hybrid ‘Zhongshanshan’.

## Materials and methods

### Sample collections and isolation

*Taxodium* hybrid ‘Zhongshanshan’ plants with dieback symptoms were observed in Jinan State-owned Beijiao Forest Farm, Jinan City, Shandong Province, China (36°46'03"N, 116°51'46"E). Bark was peeled from the sampled branches to reveal insect infestation. Larvae from the observed borings were then extracted and transported to the laboratory for identification. To isolate possible associated fungi, infected branches were excised and maintained in a humid chamber for 2–3 days to promote fungal sporulation. When white mycelium was visible, samples were transferred from 60 galleries using sterilised toothpicks on to 2% potato dextrose agar (PDA) plates, which were incubated at 25 °C for 3 days in the dark. Fungal isolates were purified with the monosporic isolation method described by Li et al. (2007). The obtained isolates were stored in the Forest Pathology Laboratory at Nanjing Forestry University (NJFU) and the China Forestry Culture Collection Center (CFCC), Chinese Academy of Forestry, Beijing, China.

### Identification of beetles

Beetles were identified through combined molecular and morphological approaches. Morphological characterisation was performed under a Zeiss stereomicroscope (SteRo Discovery v.20) with reference literature (Xiao 1992). The entire beetle larvae were placed in a 1.5 ml microcentrifuge tube for subsequent DNA extraction following the procedure described by He et al. (2021). A fragment of the cytochrome c oxidase subunit I (*COI*) gene was amplified using primers and cycling conditions following Cognato et al. (2020). Amplified products were purified and sequenced by Shanghai Sangon Biotechnology Company (Nanjing, China). Phylogenetic analysis was conducted using Maximum Likelihood (ML) in IQ-TREE (Nguyen et al. 2015). Sequence alignment was performed using the MAFFT v.7 online server (https://mafft.cbrc.jp/alignment/server/) (Katoh and Standley 2013). The best-fit nucleotide substitution model (GTR+F+I) was selected under the Akaike Information Criterion (AIC) using ModelFinder (Kalyaanamoorthy et al. 2017) as implemented in PhyloSuite (Zhang et al. 2020). Node support was assessed with 1,000 ultrafast bootstrap replicates.

### DNA extraction, PCR amplification and sequencing of fungi

Genomic DNA of all fungal isolates was extracted using a modified CTAB method (Damm et al. 2008). The fungal plugs of each isolate were grown on the PDA plates for 5 days and then collected in a 2 ml tube. Then, 500 µl of chloroform and 500 µl of hexadecyltrimethyl ammonium bromide (CTAB) extraction buffer (0.2 M Tris, 1.4 M NaCl, 20 mM EDTA, 0.2 g/l CTAB) were added into the tubes, which were placed in a shaker at 25 °C at 200 rpm for 2 h. The mixture was centrifuged at 15,800 × *g* for 5 min. Three hundred µl of the supernatant was transferred into a new tube and 600 µl of absolute ethanol (≥ 99.8%) was added. The suspension was centrifuged at 15,800 × *g* for 5 min. Then, 600 µl of 70% ethanol was added to the precipitate. The suspension was centrifuged at 15,800 × *g* for 5 min and the supernatant was discarded. The DNA pellet was dried and re-suspended in 30 µl ddH_2_O.

The polymerase chain reaction (PCR) amplification was carried out on the extracted DNA. Translation elongation factor 1-alpha (*TEF1*), RNA polymerase largest subunit (*RPB1*) and RNA polymerase second largest subunit (*RPB2*) were amplified with the primer sets of EF1/EF2 (O’Donnell et al. 1998) and Fa/G2R (O’Donnell et al. 2010) and 5f2/7cr (Liu et al. 1999), respectively. The primer sequences were listed in Suppl. material 5.

PCR was performed in a 30 μl reaction volume containing 2 μl of genomic DNA (ca. 200 ng/μl), 15 μl of 2 × Taq Plus Master Mix (Dye Plus) (Vazyme P212-01), 1 µl of 10 μM forward primer, 1 µl of 10 μM reverse primer and 11 μl of ddH_2_O. The parameters for PCR protocol were 94 °C for 4 min, followed by 34 cycles of 30 s at 94 °C, annealing at a suitable temperature for the 30 s for different loci: 55 °C for *TEF1*, 58 °C for *RPB1* and 62 °C for *RPB2*, 72 °C for 60 s and a final elongation step at 72 °C for 10 min. All DNA sequencing was performed at Shanghai Sangon Biotechnology Company (Nanjing, China). The sequences derived in this study were deposited in GenBank. GenBank accession numbers of all isolates used for phylogenetic analyses were listed in Table 1.

**Table 1.** Cultures, specimens and DNA accession numbers included in this study.

Species name	Culture/specimen^1^	Host	Country/area	GenBank/ENA accession number^2^		
				*TEF-1α*	*RPB1*	*RPB2*
***Fusarium fujikuroi* species complex**						
*F. acutatum*	CBS 402.97^T^	Unknown	India	KR071754	MT010947	KT154005
*F. agapanthi* (Outgroup)	NRRL 54463^HT^	African lily	Australia and Italy	KU900630	KU900620	KU900625
*F. agapanthi* (Outgroup)	NRRL 54464	African lily	Australia and Italy	NA	KU900622	KU900627
*F. ananatum*	CBS 118516^T^	Unknown	Unknown	NA	MT010937	KU604269
*F. annulatum*	CBS 258.54^T^	Unknown	New Caledonia	MT010994	MT010944	MT010983
*F. annulatum*	YJX-0511	tulip bulb	China	PQ791636	PQ787494	PQ791805
***F. annulatum***	SG2-21*	***Taxodium* hybrid ‘Zhongshanshan’**	**China**	**PV640581**	**PV613651**	**PV640565**
***F. annulatum***	SP2-16*	***T.* hybrid ‘Zhongshanshan’**	**China**	**PV640582**	**PV613652**	**PV640566**
*F. awaxy*	LGMF 1930^HT^	Stalk, *Zea mays*	Brazil	MG839004	NA	MK766941
*F. bactridioides*	CBS 100057^T^	*Pinus leiophylla*	Arizona	KC514053	MT010939	NA
*F. begoniae*	CBS 452.97^T^	*Begonia* elatior hybrid	Germany	KC514054	NA	MT010964
*F. brevicatenulatum*	CBS 404.97^T^	*Striga asiatica*	Madagascar	MT011005	MT010948	MT010979
*F. brevicatenulatum*	NRRL 25447	Unknown	Unknown	MN193859	NA	MN193887
*F. circinatum*	NRRL 25331^T^	Monterrey pine tree	USA	AF160295	NA	JX171623
*F. concentricum*	CBS 450.97^T^	*Musa sapientum* fruit	Costa Rica	MT010992	MT010942	MT010981
*F. concentricum*	SJ1-10	Chinese fir	China	ON734385	OR683264	ON734365
*F. concentricum*	SJ1-10-1	Chinese fir	China	ON734386	OR683265	ON734366
*F. elaeagni*	CGMCC 3.20822^T^	*Elaeagnus pungens*	China	MW580466	MW024457	MW474412
*F. elaeagni*	LC13628	*Elaeagnus pungens*	China	MW580467	MW024458	MW474413
*F. erosum*	LC18579	Maize	China	OQ126065	OQ125773	OQ126517
*F. erosum*	CGMCC3.23518^T^	Maize	China	OQ126066	OQ125772	OQ126518
*F. erosum*	LC18581	Maize	China	OQ126067	OQ125774	OQ126519
*F. fujikuroi*	MUCL 55986	*Musa* sp.	China	LT574941	NA	LT575022
*F. fujikuroi*	CBS 221.76^T^	*Oryza sativa* culm	Taiwan	KR071741	NA	KU604255
*F. fujikuroi*	HN43-17-1	Chinese fir	China	ON734397	OR683276	ON734377
***F. fujikuroi***	SG2-45*	***T.* hybrid ‘Zhongshanshan’**	**China**	**PV640583**	**PV613653**	**PV640567**
***F. fujikuroi***	SG2-27*	***T.* hybrid ‘Zhongshanshan’**	**China**	**PV640584**	**PV613654**	**PV640568**
***F. fujikuroi***	SG2-29*	***T.* hybrid ‘Zhongshanshan’**	**China**	**PV640585**	**PV613655**	**PV640569**
*F. lactis*	NRRL 25200^NT^	*Ficus carica*	USA	AF160272	MT010954	NA
*F. mangiferae*	NRRL 25226^T^	*Mangifera indica*	India	AF160281	NA	JX171622
*F. nygamai*	NRRL 13448^T^	*Necrotic sorghum root*	Australia	AF160273	MT010955	EF470114
*F. planum*	LC18541	Maize	China	OQ126127	OQ125876	OQ126557
*F. planum*	CGMCC3.23517^T^	Maize	China	OQ126125	OQ125871	OQ126555
*F. planum*	LC18411	Rice	China	OQ126124	OQ125872	OQ126561
*F. proliferatum*	F026	*Musa* sp.	China	MZ399213	MZ399206	MZ399210
*F. proliferatum*	CBS 480.96^ET^	Soil in tropical rain forest	Papua New Guinea	MN534059	NA	MN534272
*F. pseudocircinatum*	NRRL 22946^T^	*Solanum* sp.	Ghana	AF160271	MT010952	NA
*F. pseudonygamai*	NRRL 13592^T^	*Pennisetum typhoides*	Nigeria	AF160263	MT010951	NA
*F. ramigenum*	NRRL 25208^T^	*Ficus carica*	USA	AF160267	MT010959	KF466412
*F. sacchari*	NRRL 13999	*Saccharum officinarum*	India	AF160278	NA	JX171580
*F. sanyaense*	CGMCC 3.23523^T^	Maize	China	OQ126093	OQ125859	OQ126547
*F. sanyaense*	LC18540	Maize	China	OQ126095	OQ125861	OQ126549
*F. sanyaense*	LC18537	Maize	China	OQ126094	OQ125860	OQ126548
*F. subglutinans*	NRRL 22016^T^	Corn	USA	AF160289	NA	JX171599
*F. thapsinum*	NRRL 22045	*Sorghum bicolor*	South Africa	AF160270	NA	JX171600
*F. udum*	NRRL 22949	Unknown	Germany	AF160275	NA	NA
*F. xyrophilum*	NRRL 62721	*Xyris* spp.	Guyana	NA	MW402721	MN193905
*F. xyrophilum*	NRRL 62710	*Xyris* spp.	Guyana	NA	MW402720	MN193903
***F. incarnatum-equiseti* species complex**						
*F. citri*	LC13696	*Castanopsis boisii*	China	MW594366	MW024551	MW474509
*F. citri*	CGMCC 3.19467^T^	*Citrus reticulata*	China	MK289617	MK289828	MK289771
*F. compactum*	LC13699	Soil	China	MW594369	MW024554	MW474512
*F. compactum*	LC13700	*Poa annua*	China	MW594370	MW024555	MW474513
*F. equiseti*	CBS 307.94^T^	Unknown	Germany	KR071777	NA	KU604327
*F. fecundum*	CGMCC 3.23516^T^	Wheat	China	OQ125250	NA	OQ125544
*F. fecundum*	LC18376	Rice	China	OQ125249	NA	OQ125543
*F. fecundum*	LC18372	Rice	China	OQ125248	NA	OQ125542
*F. hainanense*	CGMCC 3.19478^T^	*Oryza* sp.	China	MK289581	MK289833	MK289735
*F. hainanense*	LC12161	*Musa nana*	China	MK289595	MK289832	MK289748
*F. ipomoeae*	CGMCC 3.19496^T^	*Ipomoea aquatica*	China	MK289599	NA	MK289752
*F. ipomoeae*	LC13706	*Oryza* sp.	China	MW594376	MW024561	MW474519
*F. ipomoeae*	LC13707	Soil	China	MW594377	MW024562	MW474520
***F. ipomoeae***	SG2-36*	***T.* hybrid ‘Zhongshanshan’**	**China**	**PV640586**	**PV613656**	**PV640570**
***F. ipomoeae***	SG2-24*	***T.* hybrid ‘Zhongshanshan’**	**China**	**PV640587**	**PV613657**	**PV640571**
***F. ipomoeae***	SG2-23*	***T.* hybrid ‘Zhongshanshan’**	**China**	**PV640588**	**PV613658**	**PV640572**
*F. irregulare*	LC12145	Bamboo	China	MK289582	MK289864	MK289737
*F. irregulare*	LC12146	Bamboo	China	MK289583	MK289865	MK289738
*F. jinanense*	CGMCC 3.23519^T^	Maize	China	OQ125131	NA	OQ125521
*F. jinanense*	LC18379	Maize	China	OQ125132	NA	OQ125522
*F. luffae*	CQ1038	*Humulus scandens*	China	MK289569	MK289870	MK289723
*F. luffae*	CGMCC 3.19497^T^	*Luffa aegyptiaca*	China	MK289601	MK289869	MK289754
*F. mianyangense*	CGMCC 3.23520^T^	Rice	China	OQ125232	NA	OQ125510
*F. mianyangense*	LC18549	Maize	China	OQ125226	NA	OQ125511
*F. nanum*	CGMCC 3.19498^T^	*Musa nana*	China	MK289602	MK289871	MK289755
*F. nanum*	LC1384	*Solanum lycopersicum*	Saudi Arabia	MK289611	MK289872	MK289764
*F. nanum*	LC1385	*Solanum lycopersicum*	Saudi Arabia	MK289612	MK289873	MK289765
*F. nothincarnatum*	LC18382	Wheat	China	OQ125146	NA	OQ125508
*F. nothincarnatum*	CGMCC 3.24286^T^	Rice	China	OQ125147	NA	OQ125509
*F. sulawesiense*	LC12175	*Ipomoea aquatica*	China	MK289607	MK289823	MK289760
*F. sulawesiense*	LC12178	*Syngonium auritum*	China	MK289610	MK289826	MK289763
*F. tanahbumbuense*	LC13724	*Oryza* sp.	China	MW594394	MW024579	MW474537
*F. tanahbumbuense*	LC13725	*Oryza* sp.	China	MW594395	MW024580	MW474538
*F. tanahbumbuense*	LC13726	*Digitaria* sp.	China	MW594396	MW024581	MW474539
*F. weifangense*	CGMCC 3.24285^T^	Wheat	China	OQ125107	NA	OQ125515
*F. weifangense*	LC18311	Wheat	China	OQ125108	NA	OQ125514
***F. oxysporum* species complex**						
*F. carminascens*	CPC 25800^HT^	*Zea mays*	South Africa	MH485028	NA	MH484937
*F. carminascens*	CPC 25795	*Zea mays*	South Africa	MH485027	NA	MH484936
*F. contaminatum*	CBS 114899^HT^	Pasteurized chocolate milk	Germany	MH484992	NA	MH484901
*F. contaminatum*	ZHKUCC 23-0888	*Schlumbergera truncata*	China	PQ306362	PQ468005	PQ356481
*F. cugenangense*	InaCC F983	*Musa* sp. var. Pisang Kepok	Indonesia	LS479756	LS479559	LS479307
*F. cugenangense*	InaCC F984^HT^	*Musa* sp. var. Pisang Kepok	Indonesia	LS479757	LS479560	LS479308
*F. duoseptatum*	LC13740	*Musa nana*	China	MW594326	MW024601	MW474559
*F. duoseptatum*	LC13741	*Musa nana*	China	MW594327	MW024602	MW474560
*F. fabacearum*	LC13744	*Glycine max*	China	MW594330	MW024605	NA
*F. fabacearum*	CPC 25802^HT^	*Glycine max*	South Africa	MH485030	NA	MH484939
*F. foetens*	CBS 110286^HT^	Unknown	Unknown	MT011001	MW928808	MW928825
*F. glycines*	CPC 25808^HT^	*Glycine max*	South Africa	MH485033	NA	MH484942
*F. grosmichelii*	GDGZP11-2-2	*Musa nana*	China	OL771389	OL771373	OL771381
*F. grosmichelii*	GDZJLZ11-2-1	*Musa nana*	China	OL771390	OL771374	OL771382
*F. grosmichelii*	InaCC F833^HT^	*Musa* sp. var. Pisang Awak	Indonesia	LS479744	LS479548	LS479295
*F. hexaseptatum*	InaCC F866^HT^	*Musa acuminata* var. Pisang Ambon Kuning	Indonesia	LS479805	NA	LS479359
*F. hoodiae*	CBS 132474^HT^	*Hoodia gordonii*	South Africa	MH485020	NA	MH484929
*F. kalimantanense*	InaCC F917^HT^	*Musa acuminata* var. Pisang Ambon Kuning	Indonesia	LS479690	LS479497	LS479241
*F. languescens*	CBS 645.78^HT^	*Solanum lycopersicum*	Morocco	MH484971	MW928813	MH484880
*F. libertatis*	CPC 28465^HT^	Soil	South Africa	MH485035	NA	MH484944
*F. libertatis*	CPC 25788	*Aspalathus* sp.	South Africa	MH485024	NA	MH484933
*F. nirenbergiae*	CBS 840.88^HT^	*Dianthus caryophyllus*	Netherlands	MH484978	OP486424	MH484887
*F. odoratissimum*	LC13762	*Musa* sp.	China	MW594349	MW024624	MW474582
*F. odoratissimum*	LC13763	*Musa* sp.	China	MW594350	MW024625	MW474583
*F. odoratissimum*	InaCC F822^HT^	*Musa* sp. var. Pisang Raja	Indonesia	LS479828	LS479618	LS479386
*F. odoratissimum*	InaCC F824	*Musa* sp. var. Pisang Kepok	Indonesia	LS479678	LS479486	LS479229
*F. oxysporum* (Outgroup)	LC13766	*Malus spectabilis*	China	MW594353	MW024628	MW474586
*F. oxysporum* (Outgroup)	CBS 144134^ET^	*Solanum tuberosum*	Germany	MH485044	NA	MH484953
***F. semanoti* sp. nov**.	SG2-38 = CFCC 72663^T^*	***T.* hybrid ‘Zhongshanshan’**	**China**	**PV640589**	**PV613659**	**PV640573**
***F. semanoti* sp. nov**.	SG2-38-1*	***T.* hybrid ‘Zhongshanshan’**	**China**	**PV640590**	**PV613660**	**PV640574**
***F. semanoti* sp. nov**.	SG2-38-2*	***T.* hybrid ‘Zhongshanshan’**	**China**	**PV640591**	**PV613661**	**PV640575**
***F. sambucinum* species complex**						
*F. venenatum* (Outgroup)	CBS 458.93^T^	Winter wheat	Australia	KM231942	NA	KM232382
*F. venenatum* (Outgroup)	NRRL 25413	Unknown	United Kingdom	MW233080	MW233251	MW233423
***F. solani* species complex**						
*F. ambrosium*	NRRL 22346	*Euwallacea fornicatus*	India	FJ240350	KC691587	EU329503
*F. ambrosium*	NRRL 20438	*Euwallacea fornicatus*	India	AF178332	JX171470	JX171584
*F. bataticola*	CBS 144397	*Ipomoea batatas*	United States	AF178343	MW218099	EU329509
*F. bataticola*	CBS 144398^T^	*Ipomoea batatas*	United States	AF178344	MW218100	FJ240381
*F. borneense*	CBS 145462	Bark or recently dead tree	Indonesia	AF178352	MW834213	EU329515
*F. breviconum*	CBS 203.31	Twig	Philippines	LR583599	MW218103	LR583820
*F. cucurbiticola*	CBS 410.62	*Cucurbita viciifolia*	Netherlands	DQ247640	MW834216	LR583824
*F. cucurbiticola*	CBS 616.66^T^	*Cucurbita viciifolia*	Netherlands	DQ247592	MW834217	LR583825
*F. euwallaceae*	CBS 135854^T^	*Euwallacea* sp. on *Persea americana*	Israel	JQ038007	JQ038021	JQ038028
*F. euwallaceae*	NRRL 62626	*Euwallacea* sp. on *Persea americana*	United States	KC691532	KU171682	KU171702
*F. falciforme*	CBS 475.67^T^	Human	Puerto Rico	LT906669	MW218114	LT960558
*F. falciforme*	LC13830	*Paspalum vaginatum*	China	MW620171	MW024738	MW474696
*F. hunanense*	HN33-8-2^T^	Chinese fir	China	ON734393	OR683272	ON734373
*F. hunanense*	HN33-8-2-1	Chinese fir	China	ON734394	OR683273	ON734374
*F. keratoplasticum*	CBS 490.63^T^	Human	Japan	LT906670	MW218121	LT960562
*F. keratoplasticum*	CBS 144389	Greenhouse humic soil	Belgium	LR583613	MW218122	LR583836
*F. kuroshium*	CBS 142642^T^	*Euwallacea* sp. on *Platanus racemosa*	United States	KX262216	MW834227	LR583837
*F. liriodendri*	CBS 117481^HT^	*Liriodendron tulipifera*	Unknown	HE647957	MW218124	JX435255
*F. liriodendri*	NC21953	*Canavalia ensiformis*	South Korea	OP920672	NA	OP920674
*F. oblongum*	LC7499	Carbonatite	China	MW620179	MW024746	NA
*F. oblongum*	NRRL 28008^HT^	Human cornea	USA	DQ246868	HM347148	EF470135
***F. oblongum***	SG2-14*	***T.* hybrid ‘Zhongshanshan’**	**China**	**PV640592**	**PV613662**	**PV640576**
***F. oblongum***	SG2-25*	***T.* hybrid ‘Zhongshanshan’**	**China**	**PV640593**	**PV613663**	**PV640577**
*F. oligoseptatum*	CBS 143241^T^	*Euwallacea validus* on *Ailanthus altissima*	United States	KC691538	NA	LR583854
*F. oligoseptatum*	NRRL 62578	*Euwallacea validus* on *Ailanthus altissima*	United States	KC691537	KC691595	KC691626
*F. paraeumartii*	CBS 487.76^T^	*Solanum tuberosum*	Argentina	DQ247549	MW834240	LR583855
*F. paraeumartii*	LC13836	*Castanopsis fargesii*	China	MW620181	MW024748	MW474706
*F. parceramosum*	CBS 115695^HT^	Unknown	Unknown	JX435149	NA	JX435249
*F. parceramosum*	B15	*Passiflora edulis*	China	PP480008	NA	PP480042
*F. piperis*	CBS 145470^T^	*Piper nigrum*	Brazil	AF178360	MW834241	EU329513
*F. protoensiforme*	CBS 145471^T^	Dicot tree	Venezuela	AF178334	MW834244	EU329498
*F. pseudensiforme*	CBS 130.78	*Cocos nucifera*	Indonesia	DQ247635	MW834245	LR583868
*F. pseudensiforme*	CBS 125729^T^	Dead tree	Sri Lanka	KC691555	KC691615	KC691645
*F. solani*	NRRL 32741	Human eye	USA	DQ247061	NA	EU329608
*F. solani*	NRRL 66304^ET^	*Solanum tuberosum*	Slovenia	KT313611	NA	KT313623
***F. taxodii* sp. nov**.	SG2-26 = CFCC 72664^T^*	***T.* hybrid ‘Zhongshanshan’**	**China**	**PV640594**	**PV613664**	**PV640578**
***F. taxodii* sp. nov**.	SG2-26-1*	***T.* hybrid ‘Zhongshanshan’**	**China**	**PV640595**	**PV613665**	**PV640579**
***F. taxodii* sp. nov**.	SG2-26-2*	***T.* hybrid ‘Zhongshanshan’**	**China**	**PV640596**	**PV613666**	**PV640580**
*F. yamamotoi*	CBS 144395	*Xanthoxylum piperitum* branch	Japan	AF178328	MW218112	EU329496
*F. yamamotoi*	CBS 144396^ET^	*Xanthoxylum piperitum* trunk	Japan	AF178336	MW218113	FJ240380
*Fusarium* sp.	YZU 171871	*Citrus sinensis*	China	MK370098	NA	MK370099
*Fusarium* sp.	YZU 171870	*Citrus sinensis*	China	MH423886	NA	MH423885
***F. staphyleae* species complex**						
*F. staphyleae* (Outgroup)	NRRL 22316	*Staphylea trifolia*	United States	AF178361	JX171496	EU329502

1 BBA: Biologische Bundesanstalt für Land- und Forstwirtschaft, Institut für Mikrobiologie, Berlin, Germany; CBS: Westerdijk Fungal Biodiversity Institute (WI), Utrecht, The Netherlands; CGMCC: China General Microbiological Culture Collection Center, Zhongguancun, Beijing, China; CPC: Collection of P.W. Crous, held at WI; HMAS: Herbarium Mycologicum Academiae Sinicae, Chinese Academy of Sciences, Beijing, China; InaCC: Indonesian Culture Collection, Research Center for Biology, Indonesian Institute of Sciences (LIPI) Cibinong, Indonesia; NRRL: Agricultural Research Service Culture Collection, National Center for Agricultural Utilization Research, USDA, Peoria, IL, USA; URM: the University Recife Mycology culture collection at the Universidade Federal de Pernambuco, Recife, Brazil; FRC: *Fusarium* Research Center, University Park, PA, USA; MUCL: Mycotheque de l'Universite Catholique de Louvain, Louvain-la-Neuve, Belgium; ET: Ex-epitype, LT: Ex-lectotype, NT: Ex-neotype, HT: Ex-holotype, T: Ex-type. Sequences generated in this study are indicated in bold and marked with an asterisk (*). 2 *TEF-1α*: translation elongation factor 1-alpha; *RPB2*: RNA polymerase second largest subunit; *RPB1*: RNA polymerase largest subunit; NA: Not available.

### Phylogenetic analyses

The sequences generated in this study were compared against nucleotide sequences in GenBank using BLAST to determine closely-related taxa. Alignments of different loci, including the sequences obtained from this study and sequences downloaded from GenBank, were initially performed with the MAFFT v.7 online server (https://mafft.cbrc.jp/alignment/server/) (Katoh and Standley 2013) and then manually adjusted in MEGA v. 10 (Kumar et al. 2018). The post-alignment sequences of multiple loci were concatenated in PhyloSuite software (Zhang et al. 2020). Maximum Likelihood (ML) and Bayesian Inference (BI) analyses were conducted with PhyloSuite software using IQ-TREE ver. 1.6.8 (Nguyen et al. 2015) and MrBayes v. 3.2.6 (Ronquist et al. 2012), respectively. ModelFinder was used to carry out statistical selection of best-fit models of nucleotide substitution using the corrected Akaike Information Criterion (AIC) and Bayesian Information Criterion (BIC) (Kalyaanamoorthy et al. 2017) (Suppl. material 6). For ML analyses, the default parameters were used and bootstrap support (BS) was carried out using the rapid bootstrapping algorithm with the automatic halt option. The Bayesian analyses were conducted with two parallel runs of spanning 2,000,000 to 5,000,000 generations, employing a stop rule option and a sampling frequency set to each 1,000 generations. The 50% majority rule consensus trees and posterior probability (PP) values were calculated after discarding the first 25% of the samples as burn-in. Phylogenetic trees were visualised in FigTree v. 1.4.2 (http://tree.bio.ed.ac.uk/software/figtree/) (Rambaut 2014).

Phylogenetic analyses were conducted using three-locus datasets (*RPB1*, *RPB2* and *TEF-1α*) for isolates from four *Fusarium* species complexes. *Fusarium fujikuroi* species complex phylogenetic analyses included 43 isolates representing 28 related taxa. *Fusarium oxysporum* LC13766 and *F. oxysporum* CBS 144134 (ex-epitype) were used as outgroups. *Fusarium incarnatum-equiseti* species complex phylogenetic analyses included 33 isolates representing 15 related taxa. *Fusarium venenatum* CBS 458.93 (ex-type) and *F. venenatum* NRRL 25413 were used as outgroups. *Fusarium oxysporum* species complex phylogenetic analyses included 28 isolates representing 16 related taxa. *Fusarium agapanthi* NRRL 54463 (ex-type) and *F. agapanthi* NRRL 54464 were used as outgroups. *Fusarium solani* species complex phylogenetic analyses included 37 isolates representing 21 related taxa. *Fusarium staphyleae* NRRL 22316 was used as the outgroup.

The GCPSR approach was applied to assess potential recombination amongst phylogenetically closely-related taxa, based on the multi-locus sequence data (Taylor et al. 2000). Three loci (*RPB1*, *RPB2* and *TEF-1α*) were aligned and concatenated into one dataset. The concatenated alignment was analysed in SplitsTree v.4.17.1 (Huson and Bryant 2006) using the Pairwise Homoplasy Index (PHI, Φ_w_) test to detect signals of recombination. Significant recombination was inferred when Φ_w_ < 0.05. The relationships amongst the closely-related taxa were further visualised by constructing a split network using the LogDet transformation and Splits options.

### Morphological study

One representative isolate was randomly selected from each *Fusarium* species for morphological research according to the method of Leslie and Summerell (2006). The isolates were transferred from the actively growing edge of 4-day old colony by cutting mycelial blocks (6 mm in diameter), plated on to fresh potato dextrose agar (PDA) (Crous et al. 2021), oatmeal agar (OMA) (Crous et al. 2021) and synthetic nutrient-poor agar (SNA) (Crous et al. 2021) plates and incubated at 25 °C in the dark. Alternatively, the isolates were also plated on to the stems of *T.* hybrid ‘Zhongshanshan’ or carnation leaf agar (CLA) (Crous et al. 2021) to induce sporulation when this failed on other media. The growth rate was recorded by measuring the diameter of the colonies until day 5 and the mean growth rate was calculated per day. The colony characters including colony colour, texture and pigment production were also recorded. The morphology and size of ascomata and conidiomata were studied and recorded using a Zeiss stereomicroscope (SteRo Discovery v.20). The shape, colour, septation and size of conidiophores, conidiogenous cells and conidia were observed using a ZEISS Axio Imager A2m microscope (ZEISS, Germany) with differential interference contrast (DIC) optics. At least 30 measurements per structure were performed using Carl Zeiss Axio Vision software to determine their sizes, unless no or fewer individual structures were produced.

### Pathogenicity tests

The fungal isolates SG2-26, SG2-14, SG2-36, SG2-29, SG2-38 and SP2-16 were randomly selected from the *Fusarium* species for Koch’s postulates test. One-year-old *T.* hybrid ‘Zhongshanshan’ seedlings (ground diameter: 0.8–1.2 cm; height: 60–90 cm) were stem-inoculated *in vivo* with a conidial suspension of 10^6^ conidia/ml from each isolate. A sterile needle (1 mm in diameter) was used to make 5 mm deep wounds on the stems at a 45-degree angle, penetrating to the phloem surface. A 100 µl aliquot of conidial suspension was transferred to the pinhole with a pipette. The plants with ddH_2_O were used as the control. The inoculated plants were kept in a growth chamber at approximately 28 °C and 85% relative humidity with a 12-h/12-h light/dark photoperiod for over 14 days. The experiment was conducted three times and each treatment had three replicates. Pathogens were re-isolated from the resulting lesions and identified as before-described.

## Results

### Field symptoms and fungal isolation

**Figure 1.**
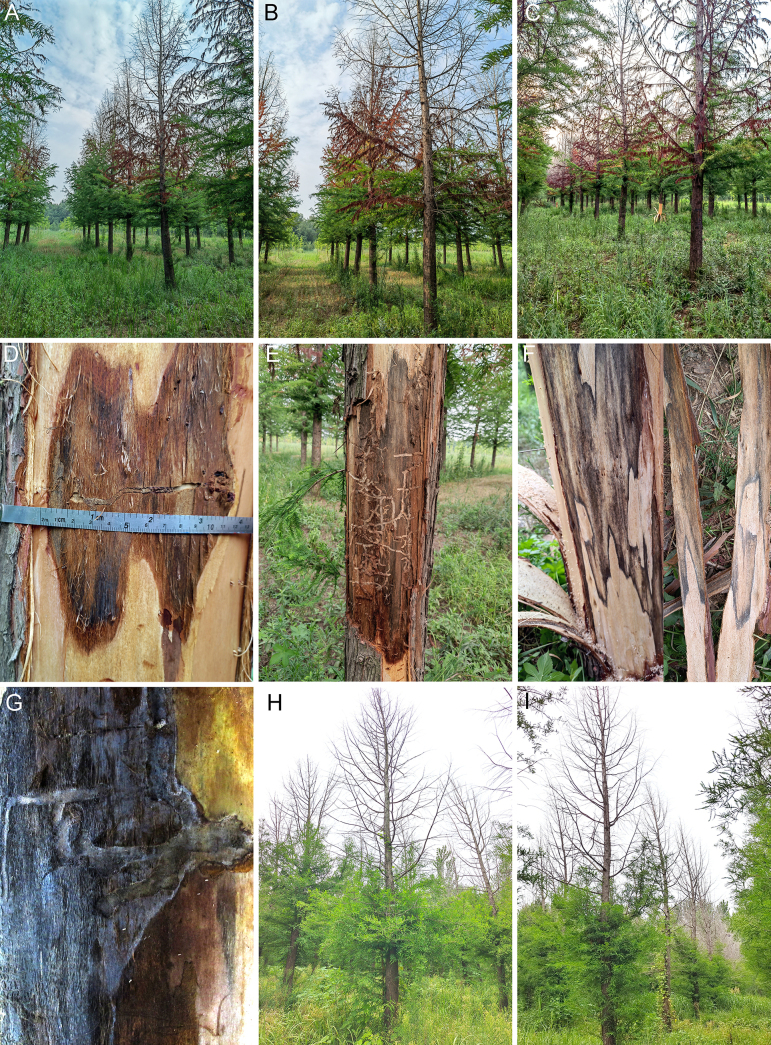
Symptoms of *Taxodium* hybrid ‘Zhongshanshan’ trees affected by *Semanotus bifasciatus* and *Fusarium* fungi. **A–C**. A *T.* hybrid ‘Zhongshanshan’ plantation with severe dieback; **D, E**. Internal symptoms of *S. bifasciatus* infestation; **F**. Black-stain in the phloem and bark; **G**. White mycelia developed on the diseased tissues after moist incubation; **H, I**. Dieback symptoms of *T.* hybrid ‘Zhongshanshan’ second-year infection.

According to field survey statistics, a total of 126 *T.* hybrid ‘Zhongshanshan’ trees were documented in the Jinan State-owned Beijiao Forest Farm (36°46'03"N, 116°51'46"E), only 23 surviving normally while the remaining 103 exhibited complete or partial mortality and the withered foliage turned brown and was retained on the tree (Fig. 1A–C). Beneath the bark, larval galleries (4–5 mm wide) were observed, with surrounding phloem softened and discoloured brown. Initially, an elliptical brown lesion (approximately 12 cm in width and 24 cm in length) developed. As insect feeding intensified, the lesion expanded progressively (Fig. 1D, E). In areas unaffected by insect activity, black discolouration was noted in both the bark and phloem (Fig. 1F). After moist incubation, white mycelia densely colonised the diseased tissues (Fig. 1G). By the following year, affected trees had either died outright or experienced extensive crown defoliation, resulting in standing dead trees (Figs 1H, 1I).

A total of 45 fungal strains were successfully isolated and purified from diseased tissue samples, of which 16 were identified as members of the *Fusarium* genus, based on morphological characteristics and ITS sequence analysis.

### Identification of beetle

**Figure 2.**
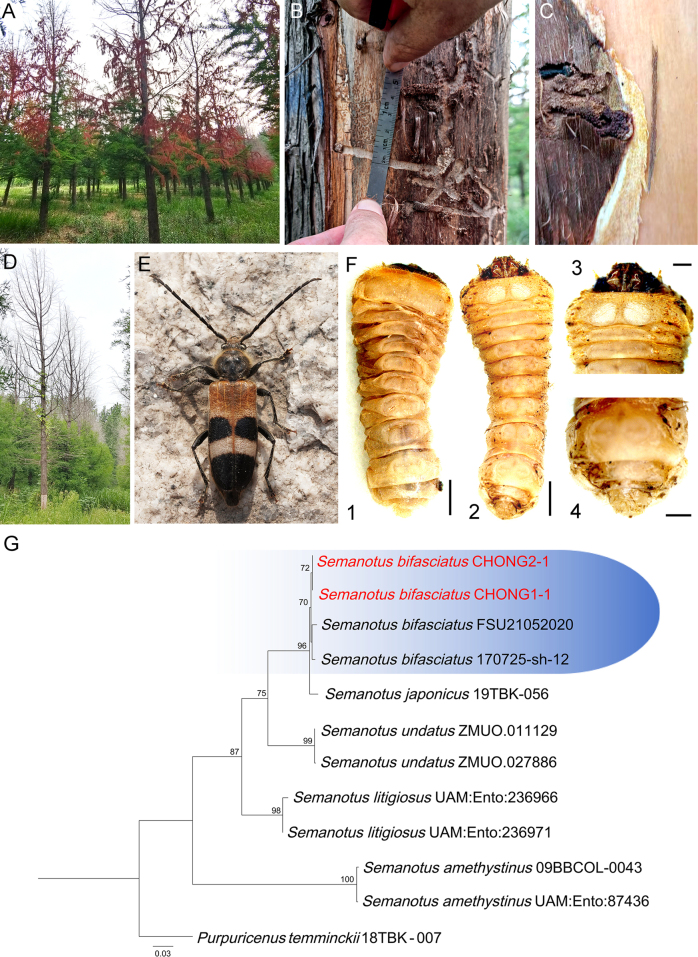
**A**. The damage symptoms of *Semanotus bifasciatus* on *Taxodium* hybrid ‘Zhongshanshan’ in the first year; **B**. The larva galleries approximately 4–5 mm in width within the phloem; **C**. The larva along with a mixture of boring dust and excrement; **D**. The field symptoms of *T.* hybrid ‘Zhongshanshan’ in the next year following infestation by *S. bifasciatus*; **E**. The longhorned beetle adult; **F**. The larva in dorsal (**1**), ventral (**2**) view, with enlarged views of the head (**3**) and tail (**4**) in ventral aspect; **G**. Phylogenetic tree based on Maximum Likelihood (ML) analysis of *COI* gene sequences for *S. bifasciatus* CHONG1-1 and *S. bifasciatus* CHONG2-1. Bootstrap support values from ML ≥ 70% are shown at nodes (ML-BS). *Purpuricenus temminckii* 18TBK-007 was the outgroup. The two specimens were sequenced in this study are indicated in red. Scale bars: 1000 μm (**F1, F2**); 500 μm (**F3, F4**).

The larva bores into the trunk of *T.* hybrid ‘Zhongshanshan’, creating galleries approximately 4–5 mm in width within the phloem. This activity leads to the dieback of branches and results in the formation of standing dead trees by the following year (Fig. 2A–D). The longhorned beetle adult discovered in the field (Fig. 2E) and two 3^rd^-instar larvae were collected from larval galleries (Fig. 2F). The morphological characteristics of both the adult and larvae align with the description of *S. bifasciatus* provided by (Xiao 1992). Furthermore, based on phylogenetic analysis employing the cytochrome c oxidase subunit I (*COI*) gene of two larvae, the insect specimens CHONG1-1 and CHONG2-1 in this study clustered in *S. bifasciatus* clade with high support values (ML-BS = 96) (Fig. 2G). The *COI* sequence homology of insect specimen CHONG1-1 with *S. bifasciatus* FSU21052020 and *S. bifasciatus* 170725-sh-12 were 99%.

### Phylogenetic analyses

**Figure 3.**
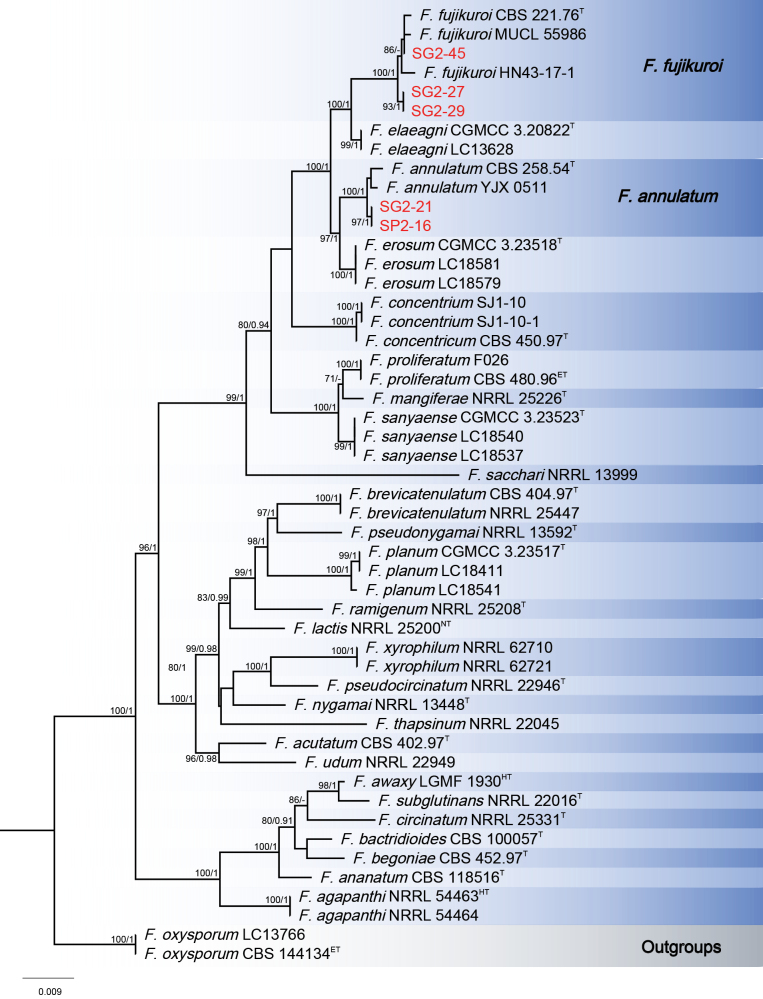
Phylogenetic relationships five isolates from the current study with 38 isolates of the *Fusarium fujikuroi* species complex derived from concatenated sequences of the *RPB1*, *RPB2* and *TEF-1α* using Bayesian Inference (BI) and Maximum Likelihood (ML) methods. Bootstrap support values from ML ≥ 70% and BI posterior values ≥ 0.9 are shown at nodes (ML/BI). *Fusarium oxysporum* LC13766 and *F. oxysporum* CBS 144134 (ex-epitype) were used as the out-groups. The strains of this study were highlighted in red. ^T^: ex-type, ^ET^: ex-epitype. ^LT^: Ex-lectotype, ^NT^: Ex-neotype, ^HT^: Ex-holotype.

A total of 16 *Fusarium* isolates were isolated from the diseased tissue samples used for phylogenetic analyses. The Bayesian Inference (BI) and Maximum-Likelihood (ML) phylogenetic analyses of the isolates of *F. fujikuroi* species complex produced topologically similar trees. The BI posterior probabilities (PP) were plotted on the ML tree (Fig. 3). In the combined analyses, three isolates (SG2-45, SG2-27 and SG2-29) clustered in *F. fujikuroi* Nirenberg clade with high support values (ML-BS/BI-PP = 100/1). Two isolates (SG2-21 and SP2-16) were placed in *F. annulatum* Bugnic. clade with high support (ML-BS/BI-PP = 100/1).

**Figure 4.**
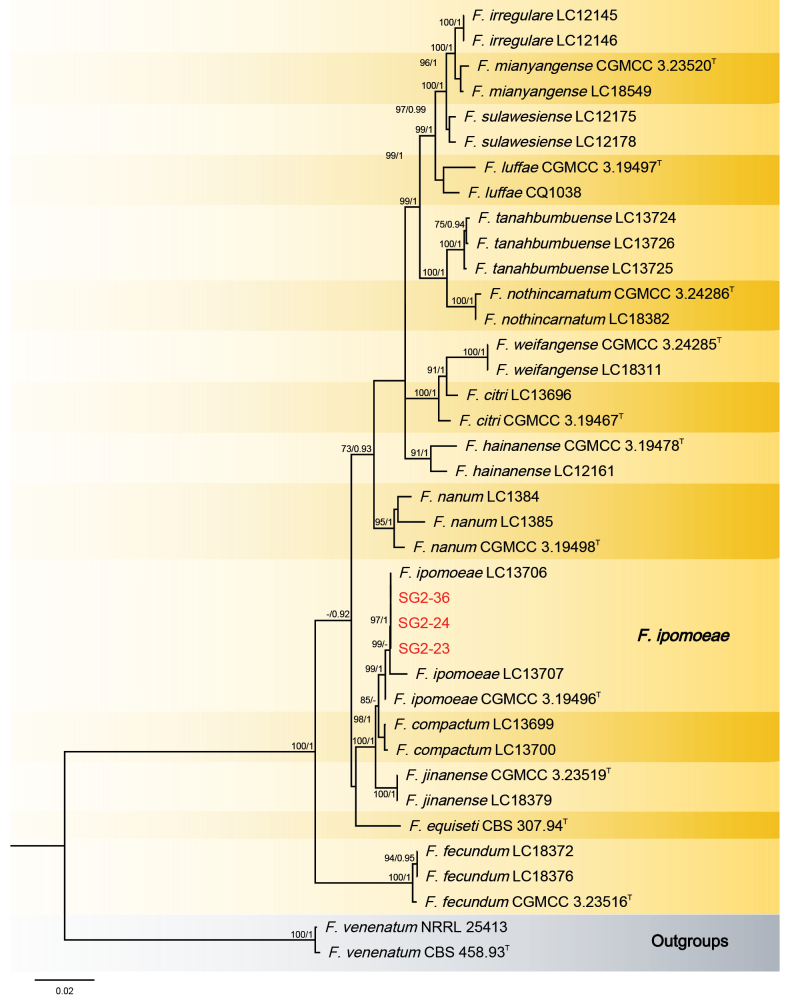
Phylogenetic relationships of three isolates from this study with 33 isolates of the *Fusarium incarnatum-equiseti* species complex derived from concatenated sequences of the *RPB1*, *RPB2* and *TEF-1α* using Bayesian Inference (BI) and Maximum Likelihood (ML) methods. Bootstrap support values from ML ≥ 70% and BI posterior values ≥ 0.9 are shown at nodes (ML/BI). *Fusarium venenatum* CBS 458.93 (ex-type) and *F. venenatum* NRRL 25413 were used as the out-groups. The strains of this study are in red. ^T^: ex-type, ^ET^: ex-epitype. ^LT^: Ex-lectotype, ^NT^: Ex-neotype, ^HT^: Ex-holotype.

The Bayesian Inference (BI) and Maximum-Likelihood (ML) phylogenetic analyses of the isolates of *F. incarnatum-equiseti* species complex produced topologically similar trees. The BI posterior probabilities (PP) were plotted on the ML tree (Fig. 4). Phylogenetic analyses showed that the three isolates (SG2-36, SG2-24 and SG2-23) clustered in one clade corresponding to *F. ipomoeae* M.M. Wang, Qian Chen & L. Cai with high support values (ML-BS/BI-PP = 99/1).

**Figure 5.**
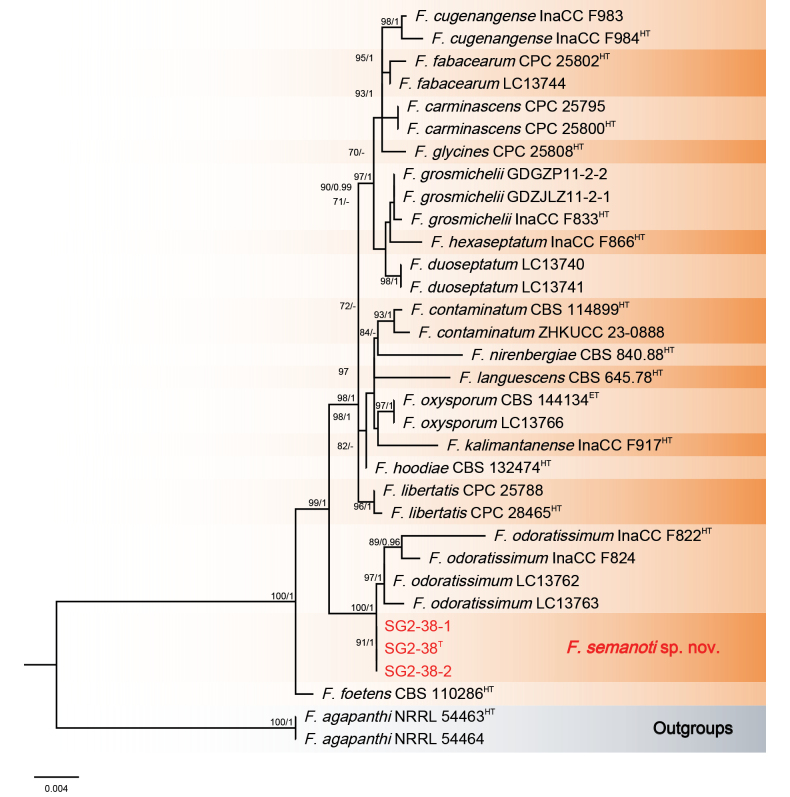
Phylogenetic relationships of *Fusarium semanoti* with 28 isolates of the *Fusarium oxysporum* species complex derived from concatenated sequences of the *RPB1*, *RPB2* and *TEF-1α* using Bayesian Inference (BI) and Maximum Likelihood (ML) methods. Bootstrap support values from ML ≥ 70% and BI posterior values ≥ 0.9 are shown at nodes (ML/BI). *Fusarium agapanthi* NRRL 54463 (ex-holotype) and *F. agapanthi* NRRL 54464 were used as the out-groups. The strains of this study are in red. ^T^: ex-type, ^ET^: ex-epitype. ^LT^: Ex-lectotype, ^NT^: Ex-neotype, ^HT^: Ex-holotype.

**Figure 6.**
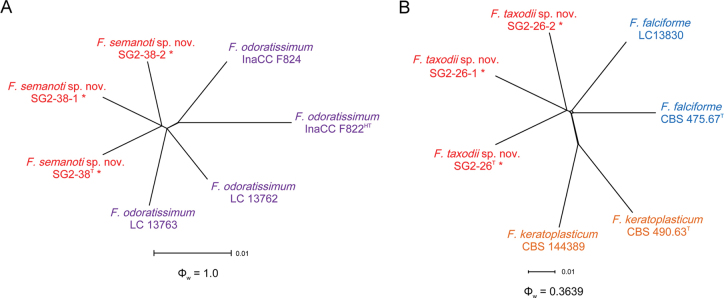
Split-graphs showing the results of the pairwise homoplasy index (PHI) test of two newly-described taxa and closely-related species using both LogDet transformation and splits decomposition. **A**. The PHI of *Fusarium semanoti* sp. nov. with their phylogenetically related isolates or species; **B**. The PHI of *F. taxodii* sp. nov. with their phylogenetically related isolates or species. PHI test value (Φ_w_) < 0.05 indicate significant recombination within a dataset. * indicates isolates of this study. ^T^ indicates ex-types. ^HT^ indicates ex-holotypes.

The Bayesian Inference (BI) and Maximum-Likelihood (ML) phylogenetic analyses of the isolates of *F. oxysporum* species complex produced topologically similar trees. The BI posterior probabilities (PP) were plotted on the ML tree (Fig. 5). Phylogenetic analyses showed that the three isolates (SG2-38, SG2-38-1 and SG2-38-2), representing a new species, clustered in the *Fusarium oxysporum* species complex, forming an independent lineage with high support values (ML-BS/BI-PP = 99/1), related to *F. odoratissimum* N. Maryani, L. Lombard, Kema & Crous. When applying the GCPSR concept to these isolates, the concatenated sequence dataset of three-loci (*RPB1*, *RPB2* and *TEF-1α*) was subjected to the PHI test and showed that no significant recombination was detected amongst these isolates/taxa (Φ_w_ = 1.0) (Fig. 6A), which was a solid support for the proposition that these isolates belonged to a distinct taxon.

**Figure 7.**
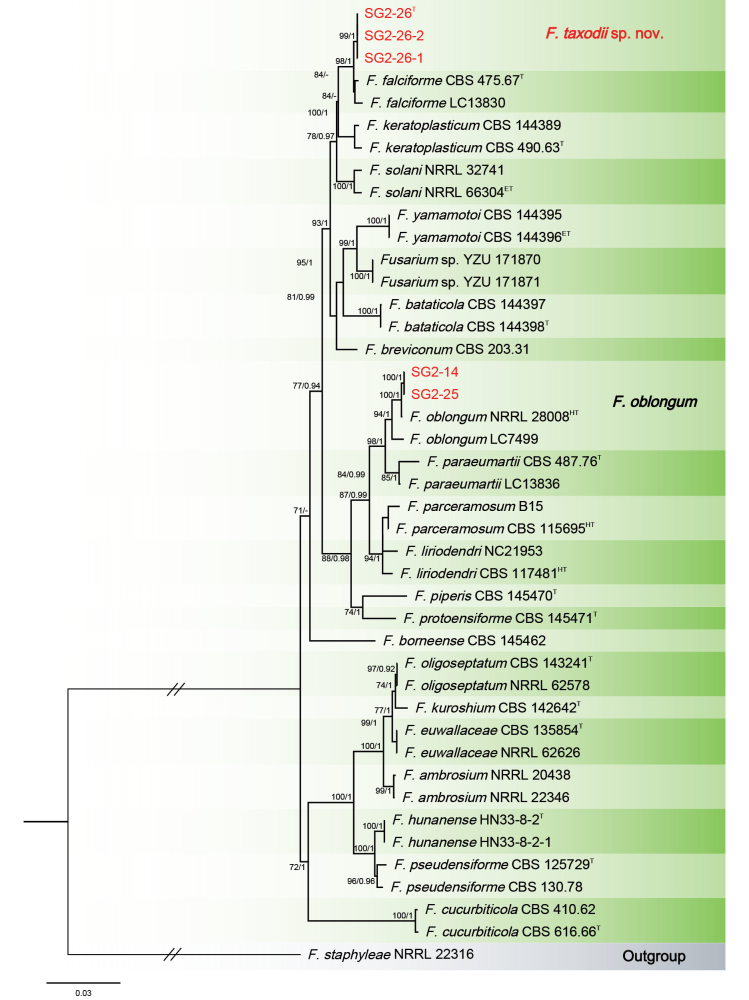
Phylogenetic relationships of *Fusarium taxodii* isolates with 37 isolates of the *Fusarium solani* species complex derived from concatenated sequences of the *RPB1*, *RPB2* and *TEF-1α* using Bayesian Inference (BI) and Maximum Likelihood (ML) methods. Bootstrap support values from ML ≥ 70% and BI posterior values ≥ 0.9 are shown at nodes (ML/BI). *Fusarium staphyleae* NRRL 22316 was used as the out-group. The strains of this study are in red. ^T^: ex-type, ^ET^: ex-epitype. ^LT^: Ex-lectotype, ^NT^: Ex-neotype, ^HT^: Ex-holotype.

The Bayesian Inference (BI) and Maximum-Likelihood (ML) phylogenetic analyses of the isolates of *F. solani* species complex produced topologically similar trees. The BI posterior probabilities (PP) were plotted on the ML tree (Fig. 7). Phylogenetic analyses showed that the three isolates (SG2-26, SG2-26-1 and SG2-26-2) formed a distinct lineage, related to *F. falciforme* (Carrión) Summerb. & Schroers with strong support values (ML-BS/BI-PP = 98/1). When applying the GCPSR concept to these isolates, the concatenated sequence dataset of three-loci (*RPB1*, *RPB2* and *TEF-1α*) was subjected to the PHI test and showed that no significant recombination was detected amongst these isolates/taxa (Φ_w_ = 0.3639) (Fig. 6B). The split tree decomposition network of these multiple combinations was clearly detected within three separate groups. In contrast, two isolates (SG2-14 and SG2-25) were placed in the *F. oblongum* (Sand.-Den. & Crous) O’Donnell & al. clade with high support values (ML-BS/BI-PP = 94/1).

### Taxonomy

The results of the molecular analyses and observations of morphological characteristics in culture indicated that the 16 *Fusarium* isolates from *T.* hybrid ‘Zhongshanshan’ belonged to six *Fusarium* species. In the present study, two new species, SG2-38 (*F. semanoti* sp. nov.) and SG2-26 (*F. taxodii* sp. nov.), were described and illustrated in detail. In addition, four new host associations were recorded for SP2-16 (*F. annulatum*), SG2-29 (*F. fujikuroi*), SG2-36 (*F. ipomoeae*), and SG2-14 (*F. oblongum*).

#### Fusarium annulatum

Taxon classificationFungiHypocrealesNectriaceae

Bugnicourt, Revue Génerale de Botanique 59: 17 (1952)

84C89EDC-EA89-5CB0-94C1-BC1BC100916C

MycoBank No: 297536

##### Host/ distribution.

From *T.* hybrid ‘Zhongshanshan’ in Jinan State-owned Beijiao Forest Farm, Jinan City, Shandong Province, China.

##### Description.

Sexual state not observed. Asexual state: Sporodochia not observed. Conidiophores borne on aerial mycelia, (8.6–)13.7–31.3(–50.3) μm, (mean ± SD = 22.5 ± 8.8 μm, n = 39), unbranched or branched, bearing terminal or intercalary monophialides, often reduced to single phialides; aerial phialides subulate to subcylindrical, smooth, thin-walled, (8.6–)10.9–19.5(–23.8) × (1.9–)2.5–3.8(–4.7) μm, (mean ± SD = 15.2 ± 4.3 × 3.2 ± 0.7 μm, n = 22), periclinal thickening inconspicuous or absent; aerial microconidia forming small false heads on tips of monophialides, hyaline, subglobose, oval, reniform or obovoid with a truncate base, ellipsoidal, smooth- and thin-walled, 0–1-septate, (6.3–)6.7–8.1(–8.7) × (1.9–)2–2.9(–3.2) μm, (mean ± SD = 7.4 ± 0.7 × 2.4 ± 0.5 μm, n = 33). Macroconidia and chlamydospores not observed.

##### Culture characteristics.

Colonies on PDA growing in the dark with an average growth rate of 10.3 mm/d at 25 °C. Colony colour white at first, becoming pale yellow, dense at the centre with radially sparse marginal mycelia. Aerial mycelium scant. Reverse pale yellow. Odour absent. Colonies on OMA incubated at 25 °C in the dark showing regular morphology, aerial mycelium sparse, covering the medium in five days. Reverse white without pigments. Colonies on SNA incubated at 25 °C in the dark exhibiting regular morphology, growing at 9.9 mm/d. Colony surface pure white, aerial mycelium scant. Reverse pure white, without diffusible pigments.

##### Materials examined.

China • Shandong province, Jinan City, Jinan State-owned Beijiao Forest Farm, 36°46'03"N, 116°51'46"E, isolated from the dead branches of *Taxodium* hybrid ‘Zhongshanshan’, August 2024, Lin Huang, isolates: SP2-16, SG2-21.

##### Notes.

Based on the results of phylogenetic analyses using multi-locus sequence data (*RPB1*, *RPB2* and *TEF-1α*), the isolate of SP2-16 in this study was in the same clade with *F. annulatum* CBS 258.54 (ex-type) (Fig. 3). Morphologically, microconidia (6.7–8.1 × 2–2.9 μm) of the isolate SP2-16 were similar to microconidia (4.7–14.4 × 1.7–2.3 μm) of the ex-type (CBS 258.54) of *F. annulatum* (Bugnicourt 1952). *Fusarium annulatum* was a pathogen in *Oryza sativa* L. in New Caledonia and former Dutch New Guinea (part of Indonesia) (Bugnicourt 1952). However, in this study, *F. annulatum* was reported for the first time as the pathogenic species of *T.* hybrid ‘Zhongshanshan’.

#### Fusarium fujikuroi

Taxon classificationFungiHypocrealesNectriaceae

Nirenberg, Mitteilungen der Biologischen Bundesanstalt für Land- und Forstwirtschaft 169: 32 (1976)

2A0614C6-35E0-5DDB-8DBF-A957F002F0EA

MycoBank No: 314213

##### Host/ distribution.

From *T.* hybrid ‘Zhongshanshan’ in Jinan State-owned Beijiao Forest Farm, Jinan City, Shandong Province, China.

##### Description.

Sexual state not observed. Asexual state: Sporodochia not observed. Conidiophores in the aerial mycelium unbranched or branched, bearing terminal or intercalary phialides. Phialides subulate to subcylindrical, smooth, thin-walled, (7.4–)11.5–17.3(–19.7) × (2.3–)2.9–3.6(–4.1) μm, (mean ± SD = 14.4 ± 2.9 × 3.3 ± 0.4 μm, n = 19), periclinal thickening inconspicuous or absent; aerial microconidia hyaline, short clavate to cylindrical, slender to relatively straight, smooth, thin-walled, 0-septate, (7.2–)8.5–10.9(–12.3) × (3–)3.7–4.6(–4.8) μm, (mean ± SD = 9.7 ± 1.2 × 4.1 ± 0.5 μm, n = 35), forming small false heads on the tips of phialides. Macroconidia and chlamydospores not observed.

##### Culture characteristics.

Colonies on PDA growing in the dark with an average growth rate of 11.3 mm/d at 25 °C. Colony colour white at first, becoming purple, felty to cottony. Aerial mycelium abundant, loose to densely floccose; margins regular and fimbriate. The reverse is bright yellow with a purple halo. Odour absent. Colonies on OMA incubated at 25 °C in the dark was regular, aerial mycelium abundant, loose to densely floccose, filling the OMA in five days. Colony in reverse was white with purple pigmentation. Colonies on SNA incubated at 25 °C in the dark was irregular, growing at 10.6 mm/d. Colony surface pure white, aerial mycelium scant, forming regular rings at the periphery of the colony. Reverse pure white, without diffusible pigments.

##### Materials examined.

China • Shandong Province, Jinan City, Jinan State-owned Beijiao Forest Farm, 36°46'03"N, 116°51'46"E, isolated from branches of *Taxodium* hybrid ‘Zhongshanshan’, August 2024, Lin Huang, isolates: SG2-27, SG2-29, SG2-45.

##### Notes.

Based on the results of phylogenetic analyses using multi-locus sequence data (*RPB1*, *RPB2* and *TEF-1α*), the isolate of SG2-29 in this study was in the same clade with *F. fujikuroi* CBS 221.76 (ex-type) (Fig. 3). Morphologically, the microconidia (8.5–10.9 × 3.7–4.6 μm) of the isolate SG2-29 were similar to the microconidia (5.0–13.5 × 2.1–4.7 μm) of the ex-type (CBS 221.76) of *F. fujikuroi* (Nirenberg 1976). *Fusarium fujikuroi* was a pathogen in *Oryza sativa* L. in Taiwan Province, China (Nirenberg 1976). However, in this study, *F. fujikuroi* was reported for the first time as the pathogenic species of *T.* hybrid ‘Zhongshanshan’.

#### Fusarium ipomoeae

Taxon classificationFungiHypocrealesNectriaceae

M.M. Wang, Q. Chen & L. Cai, Persoonia 43: 83 (2019)

396F34AE-B6CD-599E-83AB-C2EDCBDE6343

MycoBank No: 829538

##### Host/ distribution.

From *T.* hybrid ‘Zhongshanshan’ in Jinan State-owned Beijiao Forest Farm, Jinan City, Shandong Province, China.

##### Description.

Sexual state not observed. Asexual state: Sporulation abundant from sporodochia, rarely from conidiophores formed directly on the substrate mycelium. Conidiophores in the aerial mycelium absent. Sporodochia bright orange-coloured, formed abundantly on *T.* hybrid ‘Zhongshanshan’ stem. Conidiophores in sporodochia (13.1–)14.2–19.2(–22.5) μm, (mean ± SD = 16.7 ± 2.5 μm, n = 30), irregularly branched and densely packed, bearing apical whorls of 1–2 phialides; sporodochial phialides subulate to subcylindrical, smooth, thin-walled, (5.1–)6.3–11(–13.6) × (1.7–)2.4–3.6(–4.6) μm, (mean ± SD = 8.7 ± 2.3 × 3 ± 0.6 μm, n = 30). Sporodochial macroconidia colourless, falcate, with a markedly slender and nearly filiform, acicular to rat-tail-like apical cell that is frequently hooked or curved; basal cell pedicellate and distinctly stalked to inversely conical, tapering towards the point of attachment; 3–5-septate, smooth, thin-walled, constricted at the median septa, (27–)32.1–46.5(–53.8) × (3.5–)3.9–5 (–5.3) μm, (mean ± SD = 39.3 ± 7.2 × 4.5 ± 0.5 μm, n = 30). Microconidia and chlamydospores absent.

##### Culture characteristics.

Colonies on PDA growing in the dark with an average growth rate of 9.5 mm/d at 25 °C. Aerial mycelia dense, colony margin regular, surface white; the reverse is bright yellow. Odour absent. Colonies on OMA incubated at 25 °C in the dark was regular, aerial mycelium abundant, loose to densely floccose, covering the OMA in five days. Colony in reverse was white without pigmentation. Colonies on SNA incubated at 25 °C in the dark growing at 10.2 mm/d. Colony surface pure white, aerial mycelium scant, colony margin erose. Reverse pure white, without diffusible pigments.

##### Materials examined.

China • Shandong Province, Jinan City, Jinan State-owned Beijiao Forest Farm, 36°46'03"N, 116°51'46"E, isolated from branches of *Taxodium* hybrid ‘Zhongshanshan’, August 2024, Lin Huang, isolates: SG2-23, SG2-24, SG2-36.

##### Notes.

Phylogenetic analyses, based on multi-locus sequence data (*RPB1*, *RPB2* and *TEF-1α*), revealed that the SG2-36 isolate in this study clustered within the same clade as *F. ipomoeae* CGMCC 3.19496 (ex-type) (Fig. 4). Morphologically, the 3–5-septate macroconidia of isolate SG2-36 (32.1–46.5 × 3.9–5.0 μm) exhibited close similarity to those of the ex-type strain (CGMCC 3.19496) of *F. ipomoeae* (26.5–57 × 2–5 μm) as described by Wang et al. (2019). *Fusarium ipomoeae* was initially isolated from the leaves of *Ipomoea aquatica* in Fujian Province, China (Wang et al. 2019). However, in the present study, *F. ipomoeae* is reported for the first time as a pathogenic species causing dieback in *T.* hybrid ‘Zhongshanshan’.

#### Fusarium oblongum

Taxon classificationFungiHypocrealesNectriaceae

(Sandoval-Denis & Crous) O’Donnell, Geiser, Kasson & T. Aoki, mSphere 5: e00810-00820. (2020)

752B1386-C0C4-5C4D-909F-6CC2EE4235CA

MycoBank No: 831191

##### Host/ distribution.

From *T.* hybrid ‘Zhongshanshan’ in Beijiao State-Owned Forest Farm, Jinan City, Shandong Province, China.

##### Description.

Sexual state not observed. Asexual state: sporulation abundant from erect conidiophores formed on the agar surface or aggregated in sporodochia. Conidiophores in the aerial mycelium, mostly unbranched, rarely basally dichotomously branched, forming monophialides on the apices, (16.2–)36.6–89.3(–107.3) μm, (mean ± SD = 62.9 ± 26.3 μm, n = 38). Sporodochia cream-coloured, produced on the PDA medium. Conidiophores in sporodochia (19.1–)21.6–27.8(–33.6) μm, (mean ± SD = 24.7 ± 3.1 μm, n = 33), irregularly branched, short stipitate, occasionally in whorls bearing terminal 1–3 monophialides; sporodochial phialides subulate to subcylindrical, smooth, thin-walled, (7.5–)10–14(–16.4) μm × (3.3–)4.3–5.8(–6.9) μm, (mean ± SD = 12 ± 2 × 5 ± 0.8 μm, n = 30), with periclinal thickening and a small, flared collarette. Sporodochial macroconidia cylindrical to falcate, slightly curved, typically with a blunt and almost rounded apical cell and a barely notched foot cell, 3–4-septate, hyaline, smooth, thin-walled, (32.5–)39.3–46.2(–48) × (5.1–)5.7–6.7(–7.4) μm, (mean ± SD = 42.8 ± 3.4 × 6.2 ± 0.5 μm, n = 34). The microconidia were 1-celled, ovoid or ellipsoidal, hyaline, smooth and thin-walled, (4.8–)5.7–9.2(–11.8) μm × (2.3–)2.8–4.2(–5.4) μm, (mean ± SD = 7.4 ± 1.7 × 3.5 ± 0.7 μm, n = 33). Chlamydospores not observed.

##### Culture characteristics.

On PDA, the colonies were white to pale straw-coloured, aerial mycelium abundant, margins regular, velvety to finely dusty; growing in the dark with an average growth rate of 9.7 mm/d at 25 °C. Reverse pale straw to pale luteous. Odour absent. Colonies on OMA incubated at 25 °C in the dark was white, flat, velvety to dusty; aerial mycelium scant, margin regular, growing at 9.3 mm/d. Colony in reverse was white with litter grey pigmentation. Colonies on SNA incubated at 25 °C in the dark was regular, growing at 8.9 mm/d. Colony surface pure white, aerial mycelium scant. Reverse white, without diffusible pigments.

##### Materials examined.

China • Shangdong Province, Jinan City, Beijiao State-Owned Forest Farm, 36°46'03"N, 116°51'46"E, isolated from branch of *Taxodium* hybrid ‘Zhongshanshan’, August 2024, Lin Huang, isolates: SG2-14, SG2-25.

##### Notes.

Phylogenetic analyses, based on multi-locus sequence data (*RPB1*, *RPB2* and *TEF-1α*), revealed that the isolates of SG2-14 in this study clustered within the same clade as *Fusarium oblongum* (≡ *Neocosmospora oblonga*) NRRL 28008 (ex-holotype) (Fig. 7). However, the microconidia of isolate SG2-14 (5.7–9.2 × 2.8–4.2 μm) were much shorter than those of the ex-holotype (NRRL 28008) of *F. oblongum* (8–14 × 3–4 μm) (Sandoval-Denis et al. 2019; O’Donnell et al. 2020). *Fusarium oblongum* was initially isolated from the human eye in the United States (Sandoval-Denis et al. 2019; O’Donnell et al. 2020). Notably, this study represents the first report of *F. oblongum* as a pathogenic species affecting *T.* hybrid ‘Zhongshanshan’. The isolates from this study should be further studied due to their morphological difference from *Fusarium oblongum* NRRL 28008 (ex-holotype).

#### Fusarium semanoti

Taxon classificationFungiHypocrealesNectriaceae

Lin Huang, Jiao He, D.W. Li, sp . nov.

7F33C814-AB3A-5FC8-BFA1-A7CB329EC267

Index Fungorum: IF901985

Fig. 8

##### Etymology.

*semanoti*, L. insect gen. n. of *Semanotus*, referring to the association with the cedar longhorned beetle, *Semanotus bifasciatus*.

**Figure 8.**
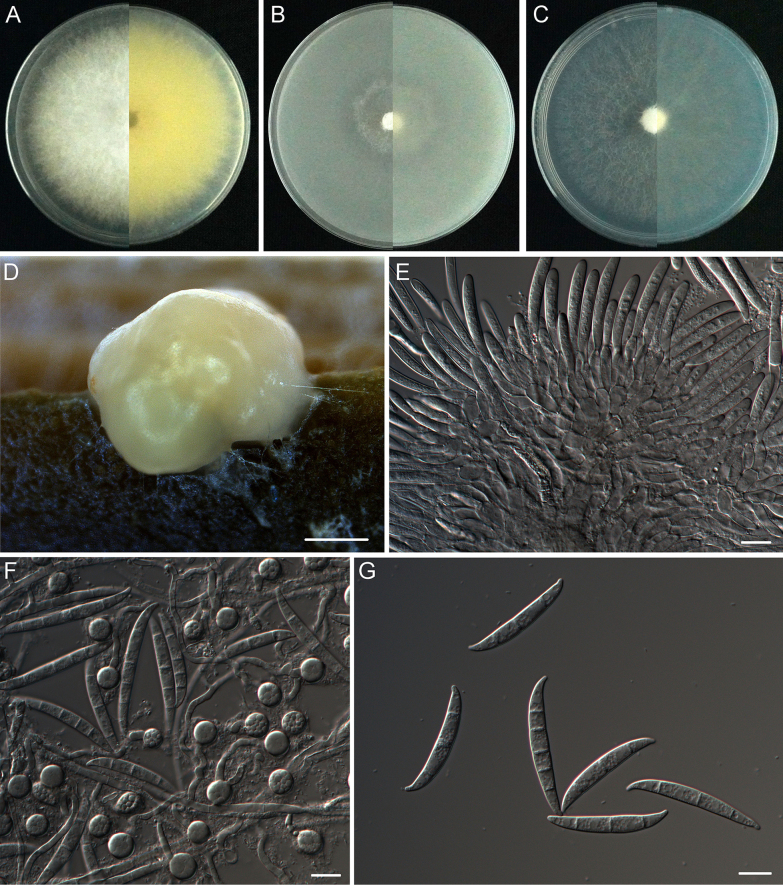
*Fusarium semanoti* sp. nov. (holotype CFCC 72663). **A–C**. Colonies on PDA, OMA and SNA, respectively, after 5 days at 25 °C in the dark; **D**. Sporodochia on the surface of carnation leaves; **E**. Sporodochial conidiophores, phialides and macroconidia; **F**. Chlamydospores and macroconidia; **G**. Macroconidia (3–5-septate). Scale bars: 500 μm (**D**); 10 μm (**E–G**).

##### Holotype.

China • Shandong Province, Jinan City, Beijiao State-owned Forest Farm, 36°46'03"N, 116°51'46"E, isolated from branch of *Taxodium* hybrid ‘Zhongshanshan’, August 2024, Lin Huang, (holotype: CFCC 72663). Holotype specimen is a living specimen being maintained via lyophilisation at the China Forestry Culture Collection Center (CFCC). Ex-type (SG3-38) is maintained at the Forest Pathology Laboratory, Nanjing Forestry University.

##### Host/ distribution.

From the *T.* hybrid ‘Zhongshanshan’ at the Beijiao State-owned Forest Farm, Jinan City, Shandong Province, China.

##### Description.

Sexual state not observed. Asexual state: sporulation abundant from sporodochia, rarely from conidiophores formed directly on the substrate mycelium. Conidiophores in the aerial mycelium absent. Sporodochia bright orange-coloured, formed abundantly on carnation leaves. Conidiophores in sporodochia (13.9–)18.1–25.4(–34.3) μm, (mean ± SD = 21.7 ± 3.6 μm, n = 36), irregularly branched and densely packed, bearing apical whorls of 1–2 phialides; sporodochial phialides subulate to subcylindrical, (6.5–)9.7–16.8(–24.6) × (3–)3.2–4.2(–4.8) μm, (mean ± SD = 13.3 ± 3.5 × 3.7 ± 0.5 μm, n = 31), smooth, thin-walled. Sporodochial macroconidia colourless, straight or slightly curved, wider at the middle or apical part, tapering towards the base, with a blunt and often curved apical cell and a foot-like to slightly notched basal cell, 3–5-septate, smooth, thin-walled, (29.9–)34.3–44.5(–48.5) × (4.3–)4.7–5.6(–6) μm, (mean ± SD = 39.4 ± 5.1 × 5.2 ± 0.4 μm, n = 35). Chlamydospores developed in large numbers in hyphae, 0-septate, globose to ellipsoidal, constricted at the septum, intercalary or terminal or solitary with mostly a pale colour and smooth, (6.7–)7.4–9.6(–11.2) × (6.1–)6.6–8.3(–9.7) μm, (mean ± SD = 8.5 ± 1.1 × 7.5 ± 0.8 μm, n = 34). Microconidia not observed.

##### Culture characteristics.

On PDA incubated at 25 °C in the dark, colonies exhibited an average radial growth rate of 11.3 mm/d. The colony surface appeared white, with a felty to cottony texture. Abundant aerial mycelium was observed, ranging from loosely to densely floccose, with regular margins and fimbriate edges. The reverse side displayed a bright yellow pigmentation. No distinctive odour. On OMA under the same conditions, colonies developed regularly, completely covering the entire 70-mm OMA plate within five days. The reverse side white with minimal grey pigmentation. On SNA incubated at 25 °C in the dark, colonies grew uniformly at 10.66 mm/d. The colony surface was pure white, with sparse aerial mycelia that formed regular peripheral rings. The reverse side remained pure white, with no observable diffusible pigments.

##### Additional materials examined.

China • Shandong Province, Jinan City, Beijiao State-owned Forest Farm, 36°46'03"N, 116°51'46"E, isolated from branch of *Taxodium* hybrid ‘Zhongshanshan’, August 2024, Lin Huang, isolates: SG2-38-1 and SG2-38-2.

##### Notes.

The isolates of *F. semanoti* were phylogenetically closely related to *F. odoratissimum* InaCC F822 (ex-holotype) (Fig. 5). Between *F. semanoti* isolates and ex-holotype of *F. odoratissimum* InaCC F822, there were 21/1432 differences in *RPB1*, 1/838 in *RPB2* and 1/573 in *TEF-1α*. The PHI analysis showed that there was no significant recombination between *F. semanoti* isolates and its related species (Φ_w_ = 1.0) (Fig. 6A). Morphological analysis revealed that macroconidia produced by isolate SG2-38 of *F. semanoti* (34.3–44.5 × 4.7–5.6 μm) were much smaller than those of the ex-holotype (InaCC F822) of *F. odoratissimum* (44–79 × 6–8 μm), as characterised by Maryani et al. (2019). Another difference is that SG2-38 of *F. semanoti* does not develop microconidia, while the ex-holotype (InaCC F822) of *F. odoratissimum* does. Thus, *F. semanoti* is recognised as a new species in *F. oxysporum* species complex.

#### Fusarium taxodii

Taxon classificationFungiHypocrealesNectriaceae

Lin Huang, Jiao He, D.W. Li, sp. nov.

8C689231-DAC7-554C-92E6-6F08BD7EEE70

Index Fungorum: IF904362

Fig. 9

##### Etymology.

The specific epithet ‘*taxodii*’ is derived from the genus name of its host plant, *Taxodium* hybrid ‘Zhongshanshan’.

**Figure 9.**
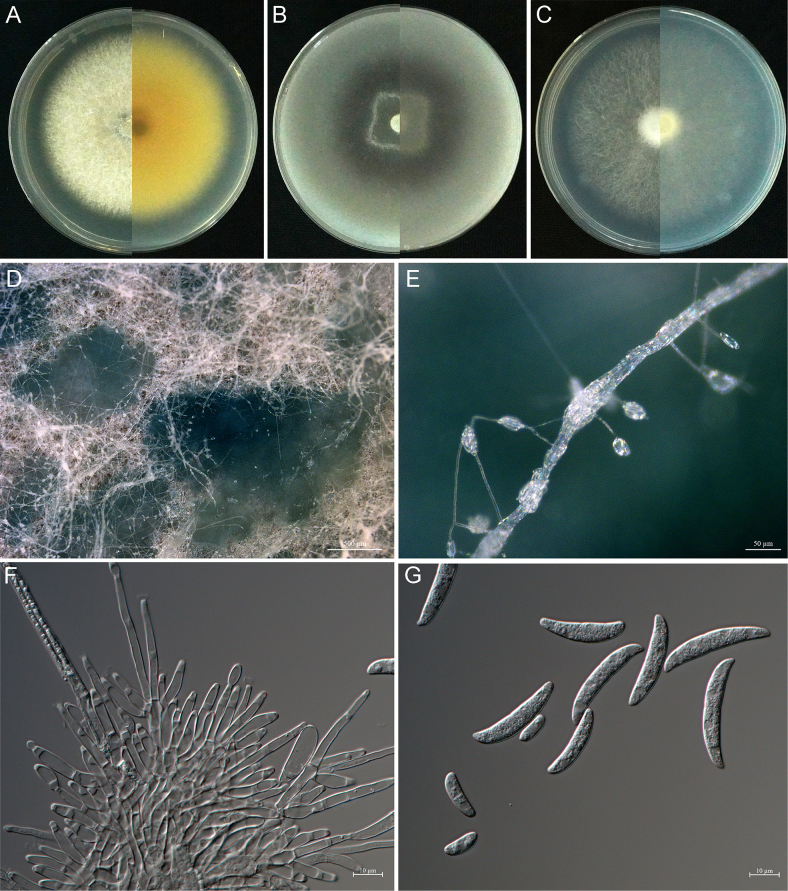
*Fusarium taxodii* sp. nov. (holotype CFCC 72664). **A–C**. Colonies on PDA, OMA and SNA, respectively, after 5 days at 24 °C in the dark; **D**. Sporodochia on PDA; **E**. Aerial conidiophores phialides and conidia on PDA; **F**. Sporodochial conidiophores and phialides; **G**. Macroconidia (2–3-septate) and microconidia (0–1-septate). Scale bars: 50 μm (**D**); 10 μm (**E–G**).

##### Holotype.

China • Shandong Province, Jinan City, Beijiao State-owned Forest Farm, 36°46'03"N, 116°51'46"E, isolated from branch of *Taxodium* hybrid ‘Zhongshanshan’, August 2024, Lin Huang, (holotype: CFCC 72664). Holotype specimen is a living specimen being maintained via lyophilisation at the China Forestry Culture Collection Center (CFCC). Ex-type (SG2-26) is maintained at the Forest Pathology Laboratory, Nanjing Forestry University.

##### Host/ distribution.

From *T.* hybrid ‘Zhongshanshan’ in Beijiao State-owned Forest Farm, Jinan City, Shandong Province, China.

##### Description.

Sexual state not observed. Asexual state: abundant sporulation produced by erect conidiophores arising from the agar surface or aggregated within sporodochia. Aerial conidiophores predominantly unbranched, with rare basal dichotomous branching, terminating in monophialides. Phialides slender, subulate to subcylindrical, monophialidic, smooth-walled, with slightly periclinal thickening at the apex and a short, flared apical collarette, (20.8–)36–72(–100.9) μm, (mean ± SD = 54 ± 18 μm, n = 33). Sporodochia bluish-grey-coloured on PDA medium. Conidiophores within sporodochia (15.6–)20.7–31.6(–37.7) μm, (mean ± SD = 26.1 ± 5.4 μm, n = 31), irregular branched, short stipes and occasional whorled arrangements bearing 1–2 terminal monophialides. Sporodochial phialides subulate to subcylindrical, smooth-walled, (8.4–)12.1–15.9(–17.2) × (3–)3.2–4.2(–4.6) μm, (mean ± SD = 14 ± 1.9 × 3.7 ± 0.5 μm, n = 32), with periclinal thickening and a small, flared collarette. Sporodochial macroconidia are cylindrical to falcate, gently curved, typically with a blunt, nearly rounded apical cell and a slightly notched foot cell, 2–3-septate, hyaline, smooth and thin-walled, (20.4–)29.6–38.4(–40.6) × (4.1–)5.4–6.9(–7.9) μm, (mean ± SD = 34 ± 4.4 × 6.1 ± 0.8 μm, n = 34). Sporodochial microconidia falcate, dorsiventrally curved, with nearly parallel sides and blunt or rounded ends, 0–1-septate, (7.2–)11.3–18.8(–22.8) × (3.9–)4.2–5.3(–6) μm, (mean ± SD = 15 ± 3.7 × 4.7 ± 0.5 μm, n = 32). Chlamydospores not observed.

##### Culture characteristics.

Colonies on PDA white, straw to buff, flat, felty to floccose; margin entire; growing in the dark with an average growth rate of 8.9 mm/d at 25 °C. Reverse white to pale straw or yellow. Odour absent. Colonies on OMA incubated at 25 °C in the dark were regular, with abundant aerial mycelium exhibiting a loose, velvety texture, covering the entire 70-mm OMA plate within five days. The colour of the colony surface and reverse was consistent, gradually transitioning from white in the centre to grey and then to greyish-white towards the edge. Colonies on SNA incubated at 25 °C in the dark were regular, growing at 9.8 mm/d. Colony surface white, aerial mycelium scant. Reverse pure white, without diffusible pigments.

##### Additional materials examined.

China • Shandong Province, Jinan City, Beijiao State-owned Forest Farm, 36°46'03"N, 116°51'46"E, isolated from trunks of *Taxodium* hybrid ‘Zhongshanshan’, August 2024, Lin Huang, isolates: SG2-26-1 and SG2-26-2.

##### Notes.

The isolates of *F. taxodii* were phylogenetically closely related to *F. falciforme* CBS 475.67 (ex-type) and *F. keratoplasticum* CBS 490.63 (ex-type) (Fig. 7). Between *F. taxodii* isolates and ex-type of *F. falciforme* CBS 475.67, there were 6/1387 differences in *RPB1*, 1/764 in *RPB2* and 7/585 in *TEF-1α*. Between *F. taxodii* isolates and ex-type of *F. keratoplasticum* CBS 490.63, there were 34/1387 differences in *RPB1*, 3/764 in *RPB2* and 9/585 in *TEF-1α*. The PHI analysis showed that there was no significant recombination amongst *F. taxodii* isolates and its related species (Φ_w_ = 0.3639) (Fig. 6B). Morphologically, microconidia (11.3–18.8 × 4.2–5.3 μm) of the isolate SG2-26 of *F. taxodii* were significantly larger than microconidia (9–12 × 3–4 μm) of the ex-type (CBS 475.67) of *F. falciforme* (Carrión 1951; Summerbell and Schroers 2002). The macroconidia (2–3-septate, 34 ± 4.4 × 6.1 ± 0.8 μm) of the isolate SG2-26 of *F. taxodii* were significantly different from macroconidia (3–5-septate, 40.1 ± 3.3 × 5.5 ± 0.2 μm) of the ex-type (CBS 490.63) of *F. keratoplasticum* (Short et al. 2013). In conclusion, the phylogenetic and morphological evidence support this fungus as being a new species within the *F. solani* species complex.

### Pathogenicity assays

**Figure 10.**
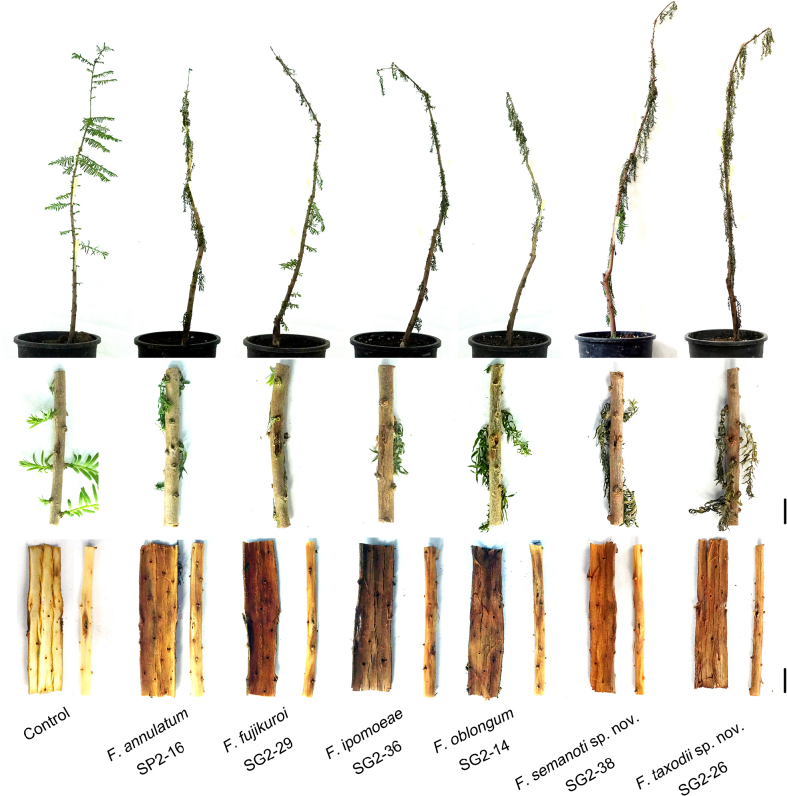
Symptoms on *T.* hybrid ‘Zhongshanshan’ stem *in vivo* at 14 days after inoculation with isolates *Fusarium annulatum* (SP2-16), *F. ipomoeae* (SG2-36) and *F. oblongum* (SG2-14); and at 28 days after inoculation with isolates *F. fujikuroi* (SG2-29), *F. semanoti* sp. nov. (SG2-38) and *F. taxodii* sp. nov. (SG2-26). Control plant was inoculated with sterile water. Scale bars: 10 mm.

Pathogenicity was tested on *T.* hybrid ‘Zhongshanshan’ stem *in vivo* following Koch’s postulates for SP2-16 (*F. annulatum*), SG2-29 (*F. fujikuroi*), SG2-36 (*F. ipomoeae*), SG2-14 (*F. oblongum*), SG2-38 (*F. semanoti* sp. nov.) and SG2-26 (*F. taxodii* sp. nov.). On the 7^th^ day post-inoculation, hyphae of isolates SP2-16 and SG2-36 had colonised the tree tissues, with white mycelia observed near the inoculation sites at the base of small branches. Isolate SG2-36 even produced orange-yellow conidial masses on the base of abscised branches. By the 14^th^ day, plants inoculated with isolates SP2-16, SG2-14 and SG2-36 exhibited wilting of the entire canopy, with drooping and easily detachable branches. Hyphal colonisation was evident within the tree tissues and, upon peeling off the bark, internal browning and softening were observed. Additionally, plants inoculated with isolates SG2-29, SG2-26 and SG2-38 did not show wilting symptoms until the 28^th^ day, indicating relatively weaker pathogenicity. In contrast, control plants inoculated with sterile water remained healthy (Fig. 10).

The fungal isolates used for inoculation were re-isolated from the diseased spots on the inoculated stems, but no fungus was isolated from the stems of the control. Therefore, Koch’s postulates were satisfied and these isolates SP2-16, SG2-29, SG2-36, SG2-38, SG2-26 and SG2-14 were determined to be the pathogens of dieback on *T.* hybrid ‘Zhongshanshan’.

## Discussion

This study investigated *T.* hybrid ‘Zhongshanshan’ damaged by *S. bifasciatus* at Jinan State-owned Beijiao Forest Farm, yielding 16 *Fusarium* isolates from beetle galleries. Through morphological and multi-locus phylogenetic analyses, we identified two new species (*F. semanoti* sp. nov. and *F. taxodii* sp. nov.) and four known species (*F. fujikuroi*, *F. annulatum*, *F. ipomoeae* and *F. oblongum*). Pathogenicity tests confirmed that all six species cause dieback in *T.* hybrid ‘Zhongshanshan’. As the first systematic study of *Fusarium* diversity associated with *S. bifasciatus* globally, this work fills a research gap while raising important questions about the ecological and functional roles of these fungi in the beetle-tree interaction.

Many species of *Fusarium* are important plant pathogens that cause wilt and other diseases in a variety of plants. In this study, we used conidial suspensions of six *Fusarium* species isolated from *S. bifasciatus* larvae galleries to inoculate the stems of *T.* hybrid ‘Zhongshanshan’. All six species induced symptoms including bark softening, branch browning and shoot tip wilting, which were consistent with the symptoms observed in naturally diseased trees in the field. These findings indicate that *Fusarium* spp. are involved in the damage process of *S. bifasciatus* on *T.* hybrid ‘Zhongshanshan’. Previous studies have demonstrated that the Asian longhorned beetle (*Anoplophora glabripennis*) can establish a close and stable symbiotic relationship with *F. solani*. When laying eggs, the beetle deposits *F. solani* along with its secretions into oviposition grooves. *Fusarium solani* then colonises these grooves and degrades lignocellulose, aiding the feeding and growth of newly-hatched larvae (Wang et al. 2023). This raises critical questions about our system: Are the *Fusarium* species identified here actively vectored by *S. bifasciatus* or do they originate from the host tree or surrounding environment? Furthermore, whether these fungi contribute to branch dieback by predisposing trees to beetle colonisation, accelerating tissue degradation or amplifying stress responses remains unclear and requires further study.

Insects and fungi have coevolved for over 400 million years, fostering diverse symbiotic relationships ranging from mutualism to antagonism (Sherwood‐Pike and Gray 1985; Biedermann and Vega 2020; Lai et al. 2022; Wang et al. 2024). Accumulating evidence underscores the pivotal role of fungal symbioses in driving forest pest outbreaks (Wingfield et al. 2016; Wingfield et al. 2017; Thakur et al. 2019). The fungi reported to be associated with longhorned beetles are primarily *Fusarium* and ophiostomatoid fungi, mainly linked to various wood-boring pests, such as *Xylotrchus rusticus* L., *Euwallacea interjectus* (Blandford), *Anoplophora glabripennis* (Motschulsky) and *Ips* bark beetles (Li et al. 2014; Lai et al. 2022; Wang et al. 2023; Wang et al. 2024). Amongst these, the symbioses between bark beetles and ophiostomatoid fungi are particularly notorious for causing huge economic and ecological damage. For example, Dutch elm disease, one of the most damaging tree diseases worldwide, is caused by *Ophiostoma ulmi* and *O. novo-ulmi*, which are vectored by scolytid bark beetles (Brasier 1991). Over the past two decades, Asian *Euwallacea*-*Fusarium* mutualists have posed a serious threat to native forests, urban landscapes and the avocado industry in Australia, Israel, Mexico and the USA (O’Donnell et al. 2016). Despite over a century of research on the associations between tree-pathogenic fungi and their insect vectors, the six *Fusarium* species identified in this study (*F. annulatum*, *F. fujikuroi*, *F. ipomoeae*, *F. oblongum*, *F. semanoti* sp. nov. and *F. taxodii* sp. nov.) have not been previously reported to be associated with other longhorned beetles. This raises intriguing questions about the specificity of the *S. bifasciatus*-*Fusarium* assemblage. Is this association unique to this beetle species or has it been overlooked in previous studies? Comparative investigations across geographically distinct *S. bifasciatus* populations and related cerambycid species are needed to address these questions.

The presence of both established pathogens (e.g. *F. fujikuroi* and *F. annulatum*, known to cause diseases in agricultural crops) and novel species within the same beetle system highlight the ecological versatility of *Fusarium*. Their ability to colonise both herbaceous and woody hosts suggests broader host adaptability than previously recognised, with implications for understanding *Fusarium* niche expansion in forest ecosystems. The discovery of two new species further emphasises that insect-associated microhabitats remain underexplored reservoirs of fungal diversity, warranting continued taxonomic surveys in understudied systems.

In conclusion, this study provides the first evidence that *S. bifasciatus* acting in synergy with six *Fusarium* species, including two new species (*F. semanoti* sp. nov. and *F. taxodii* sp. nov.) and four new host recorded species (*F. annulatum*, *F. fujikuroi*, *F. ipomoeae* and *F. oblongum*), causes extensive dieback of *T.* hybrid ‘Zhongshanshan’ in forest plantations in Jinan, Shandong Province. The spread of *S. bifasciatus* carrying these pathogenic *Fusarium* species could pose a potential threat to afforestation species, including Cupressaceae plants and to ancient tree resources. However, current research on *Fusarium* species associated with *S. bifasciatus* has been confined to localised areas in Jinan and lacks taxonomic and ecological investigations on a global scale. Furthermore, climate change may enhance the fitness, reproductive capacity and dispersal and distribution of *S. bifasciatus*, as well as the activities of the *Fusarium* species. Human-mediated long-distance invasion of either these symbiotic partners – or the organisms individually – could further exacerbate the risks. Addressing these challenges requires a multifaceted approach, including large-scale national monitoring, in-depth mechanistic research, enhanced quarantine and international cooperation and optimised silvicultural and biological control strategies, such as introducing natural enemies of the longhorned beetle, for instance, *Sclerodermus* spp. and antagonistic microbes against *Fusarium*, such as *Trichoderma* spp. (Fu et al. 2021). These actions are essential to mitigate the forest damage risks posed by this *Fusarium*-insect symbiotic system.

## Supplementary Material

XML Treatment for Fusarium annulatum

XML Treatment for Fusarium fujikuroi

XML Treatment for Fusarium ipomoeae

XML Treatment for Fusarium oblongum

XML Treatment for Fusarium semanoti

XML Treatment for Fusarium taxodii

## Additional information

### Use of AI

No use of AI was reported.

### Author ORCIDs

Jiao He https://orcid.org/0000-0002-4146-2223

Ning Li https://orcid.org/0009-0008-1414-1863

De-Wei Li https://orcid.org/0000-0002-2788-7938

Lin Huang https://orcid.org/0000-0001-7536-0914

### Data availability

The data that support the findings of this study are available from the corresponding author upon reasonable request.

## Supplementary materials

Supplementary material 1
Fusarium annulatum
Data type jpg Explanation note **fig. S1**. *Fusarium annulatum* (SP2-16). https://binary.pensoft.net/file/1582641This dataset is made available under the Open Database License (http://opendatacommons.org/licenses/odbl/1.0/). The Open Database License (ODbL) is a license agreement intended to allow users to freely share, modify, and use this Dataset while maintaining this same freedom for others, provided that the original source and author(s) are credited. Jiao He, Ning Li, De-Wei Li, Ying Guo, Shan-Shan Chen, Feng Sun, Jun-Wei Wang, Lin Huang

Supplementary material 2
Fusarium fujikuroi
Data type jpg Explanation note **fig. S2**. *Fusarium fujikuroi* (SG2-29). https://binary.pensoft.net/file/1582642This dataset is made available under the Open Database License (http://opendatacommons.org/licenses/odbl/1.0/). The Open Database License (ODbL) is a license agreement intended to allow users to freely share, modify, and use this Dataset while maintaining this same freedom for others, provided that the original source and author(s) are credited. Jiao He, Ning Li, De-Wei Li, Ying Guo, Shan-Shan Chen, Feng Sun, Jun-Wei Wang, Lin Huang

Supplementary material 3
Fusarium ipomoeae
Data type jpg Explanation note **fig. S3**. *Fusarium ipomoeae* (SG2-36). https://binary.pensoft.net/file/1582643This dataset is made available under the Open Database License (http://opendatacommons.org/licenses/odbl/1.0/). The Open Database License (ODbL) is a license agreement intended to allow users to freely share, modify, and use this Dataset while maintaining this same freedom for others, provided that the original source and author(s) are credited. Jiao He, Ning Li, De-Wei Li, Ying Guo, Shan-Shan Chen, Feng Sun, Jun-Wei Wang, Lin Huang

Supplementary material 4
Fusarium oblongum
Data type jpg Explanation note **fig. S4**. *Fusarium oblongum* (SG2-14). https://binary.pensoft.net/file/1582644This dataset is made available under the Open Database License (http://opendatacommons.org/licenses/odbl/1.0/). The Open Database License (ODbL) is a license agreement intended to allow users to freely share, modify, and use this Dataset while maintaining this same freedom for others, provided that the original source and author(s) are credited. Jiao He, Ning Li, De-Wei Li, Ying Guo, Shan-Shan Chen, Feng Sun, Jun-Wei Wang, Lin Huang

Supplementary material 5 Genes/region and respective primer pairs Data type xlsx Explanation note **table SS1**. Genes/region and respective primer pairs used in the study. https://binary.pensoft.net/file/1582645This dataset is made available under the Open Database License (http://opendatacommons.org/licenses/odbl/1.0/). The Open Database License (ODbL) is a license agreement intended to allow users to freely share, modify, and use this Dataset while maintaining this same freedom for others, provided that the original source and author(s) are credited. Jiao He, Ning Li, De-Wei Li, Ying Guo, Shan-Shan Chen, Feng Sun, Jun-Wei Wang, Lin Huang

Supplementary material 6 Nucleotide substitution models Data type xlsx Explanation note **table S2**. Nucleotide substitution models used in the phylogenetic analyses. https://binary.pensoft.net/file/1582646This dataset is made available under the Open Database License (http://opendatacommons.org/licenses/odbl/1.0/). The Open Database License (ODbL) is a license agreement intended to allow users to freely share, modify, and use this Dataset while maintaining this same freedom for others, provided that the original source and author(s) are credited. Jiao He, Ning Li, De-Wei Li, Ying Guo, Shan-Shan Chen, Feng Sun, Jun-Wei Wang, Lin Huang
