# Supplementary figures and images for: Two new species and four new host records of Fusarium species (Nectriaceae, Hypocreales) associated with Semanotus bifasciatus causing Taxodium hybrid ‘Zhongshanshan’ dieback
Source: MycoKeys. 2026 Apr 6;130:355–91. doi: 10.3897/mycokeys.130.177103 (PMC13077318; doi:10.3897/mycokeys.130.177103)

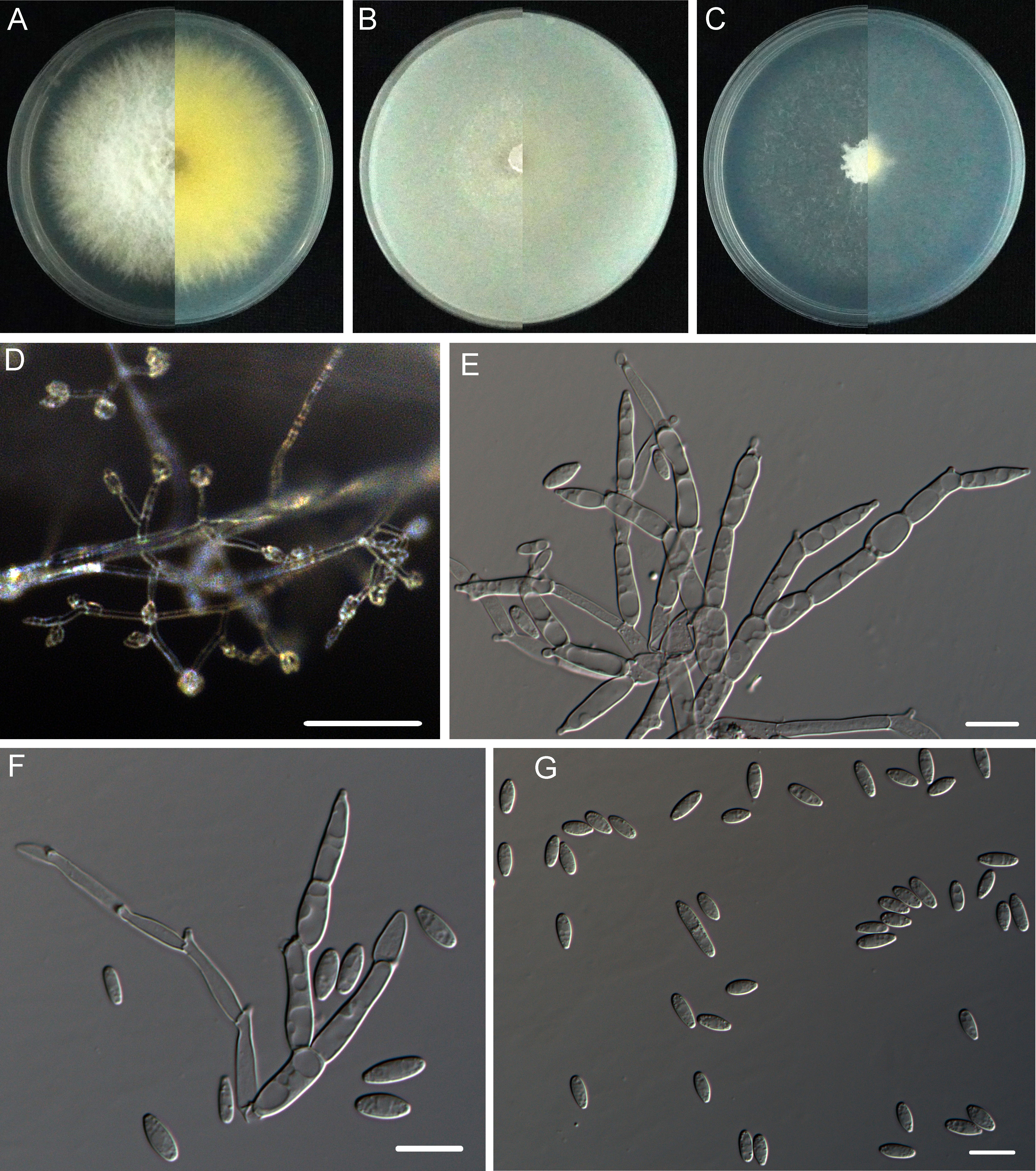

Supplement: Supplementary material 1 — Fusarium annulatum [file mycokeys-130-355-s001.jpg]

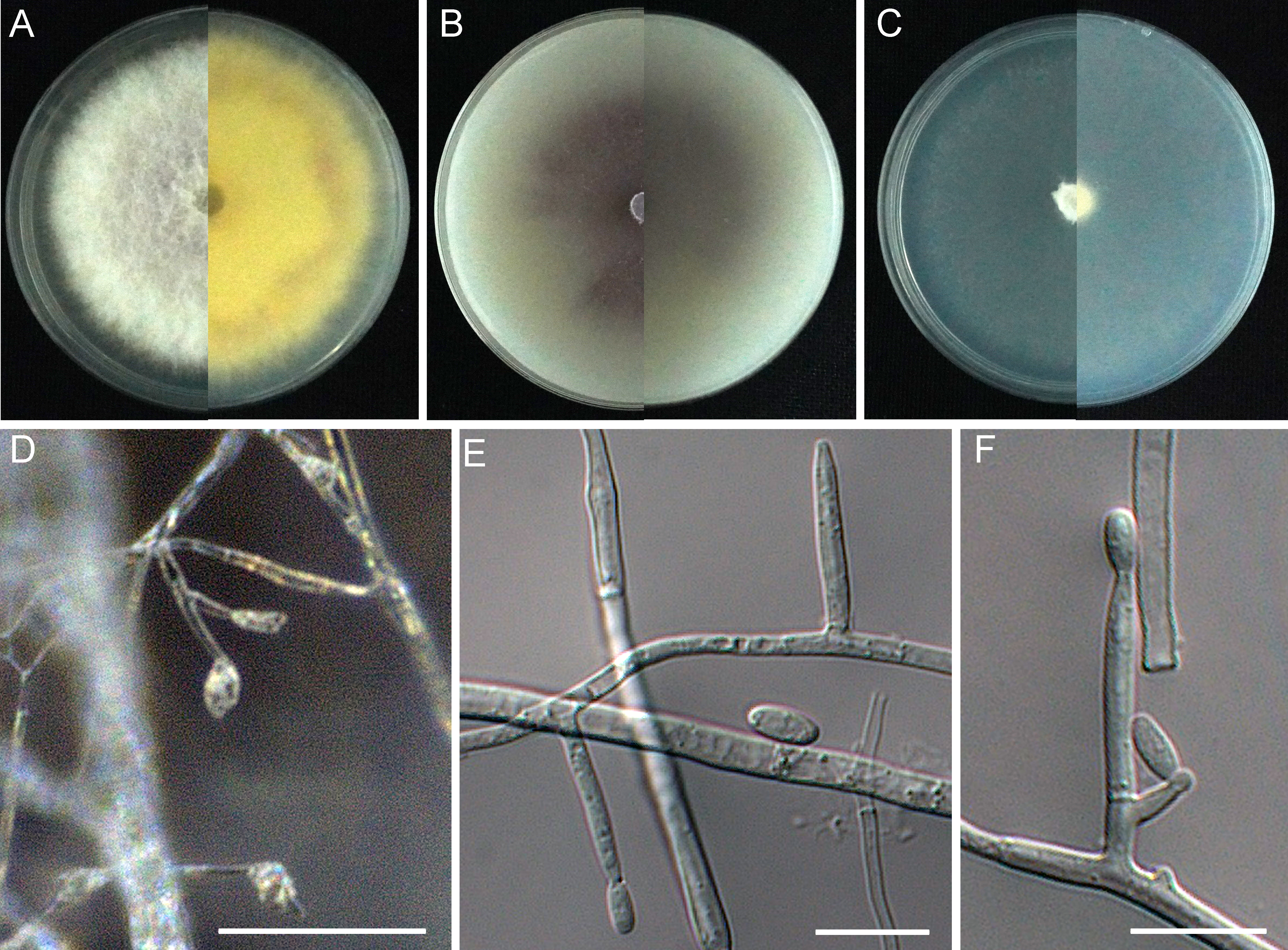

Supplement: Supplementary material 2 — Fusarium fujikuroi [file mycokeys-130-355-s002.jpg]

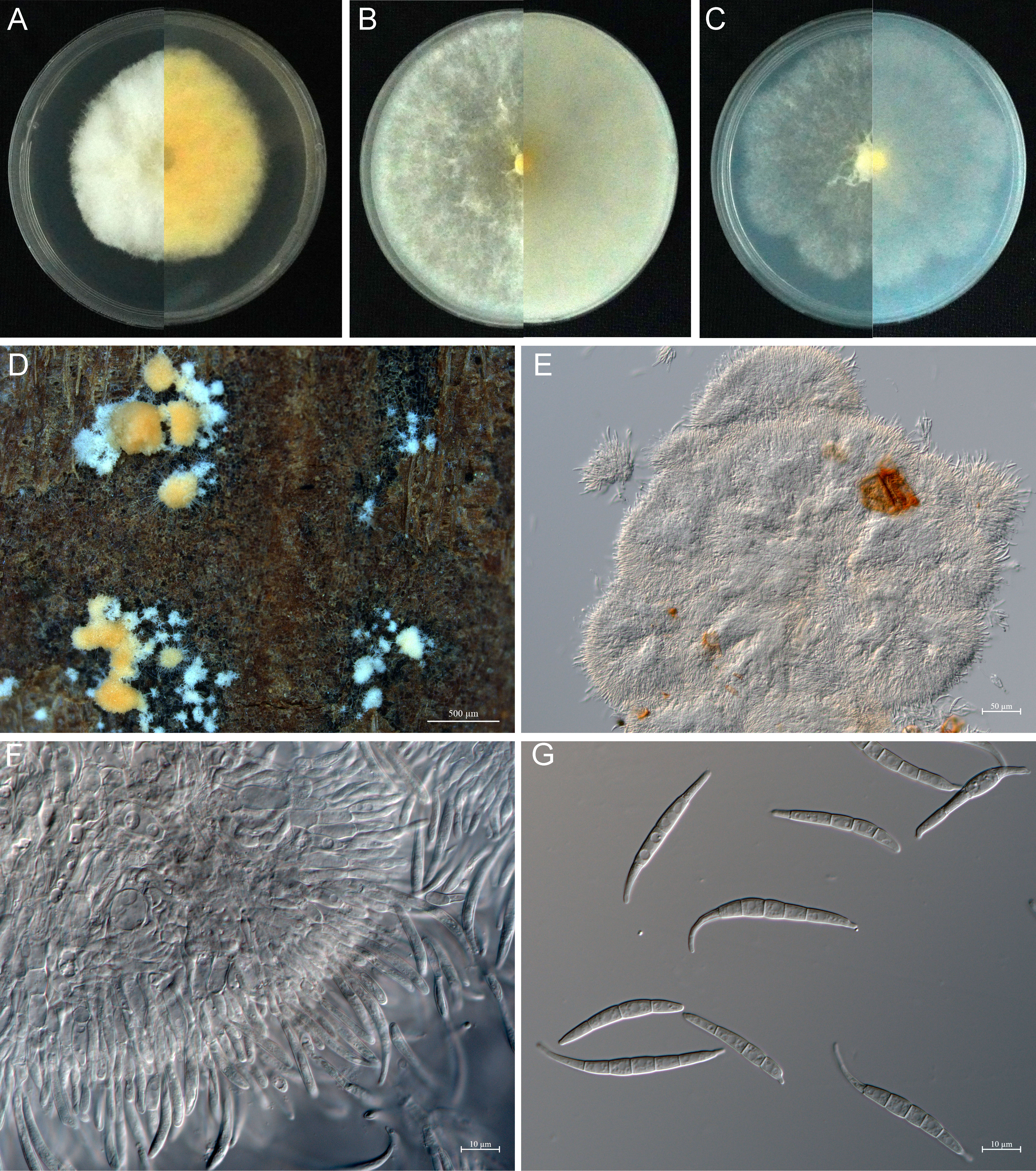

Supplement: Supplementary material 3 — Fusarium ipomoeae [file mycokeys-130-355-s003.jpg]

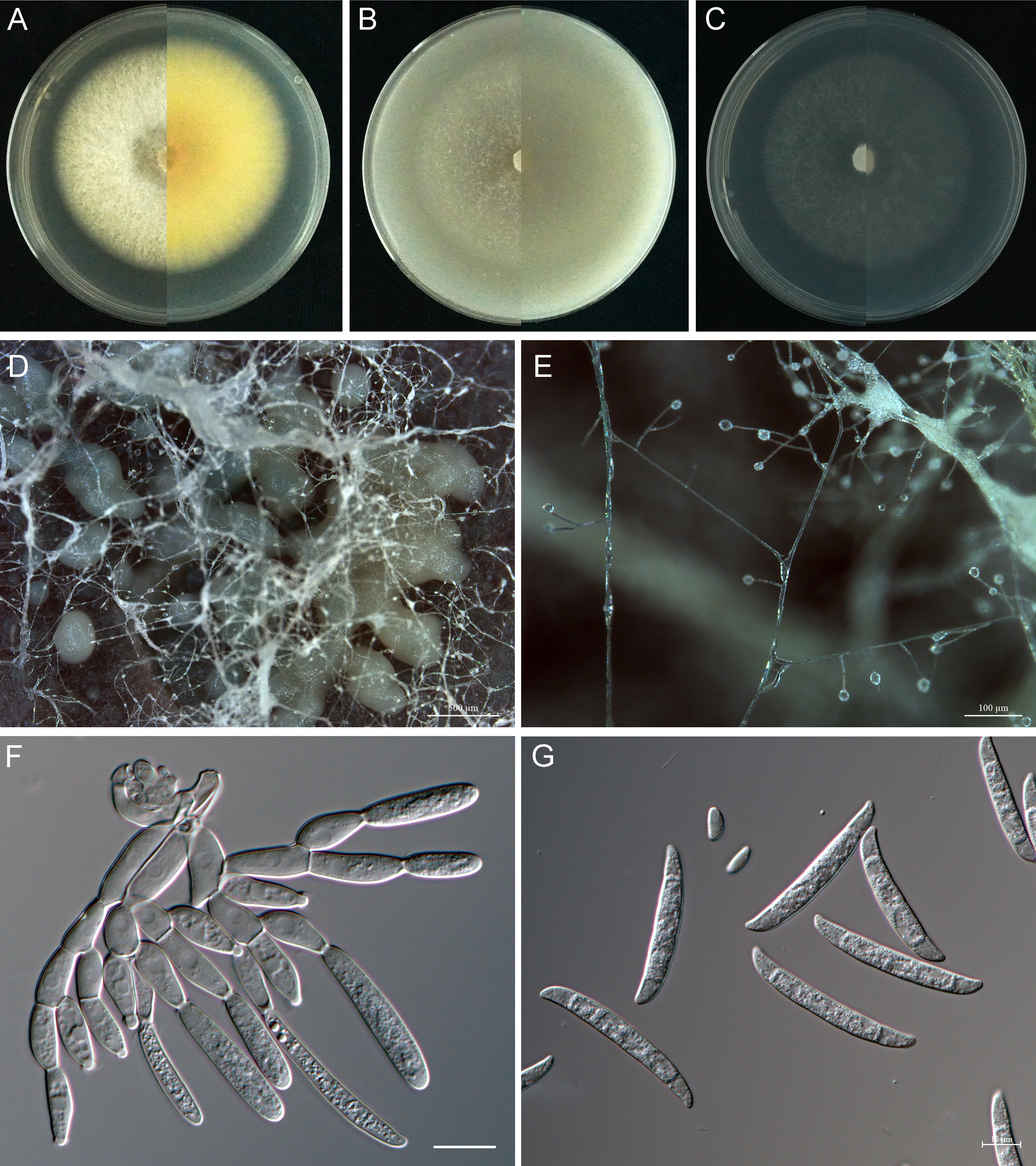

Supplement: Supplementary material 4 — Fusarium oblongum [file mycokeys-130-355-s004.jpg]
